# Use of Protamine in Nanopharmaceuticals—A Review

**DOI:** 10.3390/nano11061508

**Published:** 2021-06-07

**Authors:** Ivana Ruseska, Katja Fresacher, Christina Petschacher, Andreas Zimmer

**Affiliations:** Department of Pharmaceutical Technology and Biopharmacy, Institute of Pharmaceutical Sciences, Karl-Franzens-University Graz, Universitätsplatz 1, 8010 Graz, Austria; ivana.ruseska@uni-graz.at (I.R.); katja.fresacher@uni-graz.at (K.F.); christina.petschacher@uni-graz.at (C.P.)

**Keywords:** protamine, proticles, nanoparticles, novel vaccine technologies

## Abstract

Macromolecular biomolecules are currently dethroning classical small molecule therapeutics because of their improved targeting and delivery properties. Protamine-a small polycationic peptide-represents a promising candidate. In nature, it binds and protects DNA against degradation during spermatogenesis due to electrostatic interactions between the negatively charged DNA-phosphate backbone and the positively charged protamine. Researchers are mimicking this technique to develop innovative nanopharmaceutical drug delivery systems, incorporating protamine as a carrier for biologically active components such as DNA or RNA. The first part of this review highlights ongoing investigations in the field of protamine-associated nanotechnology, discussing the self-assembling manufacturing process and nanoparticle engineering. Immune-modulating properties of protamine are those that lead to the second key part, which is protamine in novel vaccine technologies. Protamine-based RNA delivery systems in vaccines (some belong to the new class of mRNA-vaccines) against infectious disease and their use in cancer treatment are reviewed, and we provide an update on the current state of latest developments with protamine as pharmaceutical excipient for vaccines.

## 1. Introduction

Protamines are a group of polycationic peptides present in spermatids of many animals and plants. Their history started with the discovery of the water-soluble protamine Salmine, extracted from the sperm of salmons in 1874 by Friedrich Miescher. In those days, protamines were already recognized to form insoluble salts with nucleic acids in the sperm [1,2]. Some years later, several other protamines were discovered, like Clupeine in the sperm of herrings and Scombrine in the sperm of mackerels [3]. Today, we know that two protamines, P1 and P2, can also be found in mammals. These two are said to be the most studied protamines thus far [4].

At the end of the 19th century, Albrecht Kossel proved that all protamines have one thing in common: they are strongly basic proteins, containing a high amount of the amino acid arginine (up to 70%) assembled in clusters [3,5,6]. Most of the protamines show a molecular weight of 4000–5000 Da. They are rather short proteins, comprising 50–110 amino acids and are classified into three groups according to the number of different kinds of basic amino acids they include. Monoprotamines exhibit a very simple amino acid composition incorporating only arginine as basic amino acid. Diprotamines additionally contain either the basic amino acid lysine or histidine, and triprotamines include all of the three basic amino acids [6]. The basic amino acid clusters, especially the arginine residues, represent the DNA-binding domains of protamines. These enable the formation of DNA-protamine-complexes, leading to condensation and stabilization of the spermatid genome. Protamines replace histones in this function during spermatid maturation and protect the DNA from degradation. These DNA-protamine-complexes are held together by an electrostatic linkage between the negatively charged phosphate ions of the nucleic acids and the cationic arginine moieties of protamine. The complexes are soluble in high salt concentration and show a minimum solubility in isotonic salt solutions [4,6].

At the beginning of this review, information about the structure and function of protamines, aiming especially on the mammalian protamines P1 and P2 [4,7], is summarized and protamine derivatives like protamine sulfate [8] and low-molecular-weight protamine (LMWP) [9] are discussed. After providing an introduction into the nature of protamines, one question inevitably arises: what are the main application fields of protamines? The primary use of protamines is settled in the field of medicine and pharmacy, which builds the central focus of this review. For many years, protamines are established as adjuvants in insulin preparations to prolong their effect by the complexation of insulin due to electrostatic interaction [10,11]. Additionally, protamines are used as an antidote against the anticoagulation effect of negatively charged heparin, again by building complexes with it [12,13,14,15]. 

**Figure 1:**
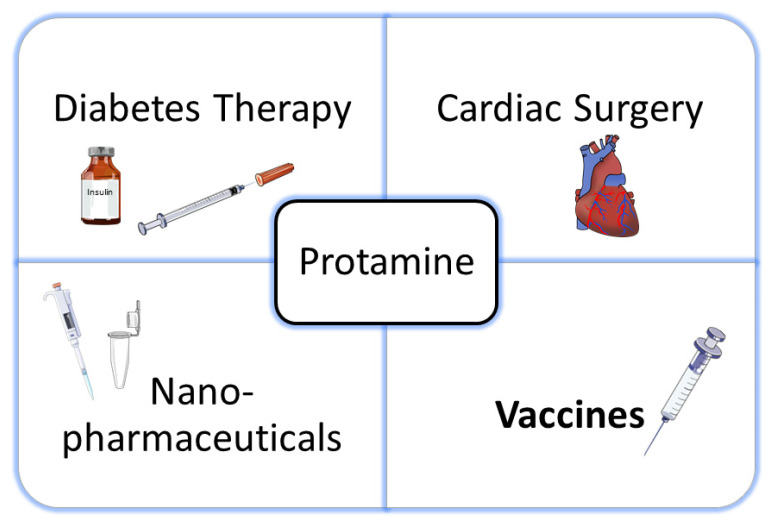
Application fields of protamine. This review will especially focus on protamine in nanopharmaceuticals as well as its approach in vaccines.

After addressing these longstanding applications of protamine (Table 1), we will focus on the use of protamine as part of drug delivery systems (Figure 1). Protamines are noninvasive cell-penetrating peptides, showing the ability to target drugs to specific molecules within the cells [9,16,17,18,19,20,21,22]. Their penetration and targeting effect can be further enhanced by creating innovative, nanosized drug delivery systems [23,24,25,26,27,28,29]. 

Thus, the first part of this review will especially highlight the ongoing research in the field of protamine-associated nanotechnology, giving details about the self-assembling manufacturing processes, the properties of the resulting nanoparticles and how they can be functionalized. 

The second key part of this review comprises a currently highly topical application field of protamines: their use as RNA-delivery systems in vaccines against infectious diseases and in cancer treatment. The outbreak of the COVID-19 pandemic at the beginning of 2020 demanded a quick development of vaccines. Today, about one year later, several vaccines against this disease are already approved and on the market. Some of them belong to the rather new class of mRNA-vaccines [30]. Due to the prevailing great interest in the subject of immunization, the second part of this review will take this topic further and opens with a general insight into the human immune system, consisting of innate and adaptive system, and its response to vaccinations, which is strongly connected to the recognition of the antigen by Toll-like receptors found on or in cells of the innate system [31,32,33].

Shedding light on new vaccine technologies, the history of vaccinology is important for the understanding of the developments in this area, namely the use of adjuvants, that increase the body’s immune response to vaccinations, and the invention of various vaccine delivery systems. Adjuvants, which are classified into immune potentiators and delivery systems, follow different mechanisms of action presented hereinafter [33,34]. The class of delivery systems is not only boosting the immune reaction but also shows important antigen transport functions.

Nanoparticles have been proven to be valuable carrier systems in vaccines, increasing their efficacy, protecting the antigen and controlling its release [33,35,36]. Liposomes, virus-like particles, polymeric nanoparticles and cell-penetrating peptides are intensively researched for this purpose [37,38,39,40], leading us back on the cell penetrating peptide protamine. Reviewing its potential in vaccine development, successful use of protamine has been published in several research articles about vaccination against infectious diseases and cancer. Giving a foretaste of this final part of the review, nanoparticles, consisting of protamine and antigen-encoding mRNA, evidentially created an immune response against the antigen after injection [41,42], and improved cell uptake was observed for protamine-antigen nanocapsules [43]. Enhanced immunogenic activity [44] as well as sustained release of the antigen was shown for protamine-antigen nanoparticles and nanocapsules, respectively [43,45,46]. Furthermore, protamine nanocarriers for vaccines revealed potential for nasal application [47,48,49] and increased thermostability [50]. 

With respect to its multiple advantageous effects as excipient in pharmaceutical preparations as summarized in the present review, protamine has proven to be a potent and versatile additive in several pharmaceutical application fields in recent decades and presents an attractive adjuvant to be considered in future research work.

## 2. Protamine-Structural Features and Function

### 2.1. Structural Features

Nearly all existing structural details of protamines and protamine–DNA complexes have been obtained from the fish protamines Salmine and Clupeine as well as from placental mammal protamines P1 and P2. A typical P1 protamine molecule comprises 49 or 50 amino acids and presents three domains: in the center is an arginine-rich DNA-binding domain flanked by short peptide chains containing cysteine residues. The amount of cysteine residues can show divergences from species to species. In general, the central DNA-binding domains comprise series of anchoring sequences, including 3–11 consecutive arginine residues to facilitate peptide-DNA binding. These special sequences show similarities in size and composition to the entire sequence of several fish protamines [4]. A more detailed description about their structures and genomes is given elsewhere [7,57]. It seems that protamine P1 and P2 are derived from one common ancestral precursor molecule but there are some features that distinguish protamine P2 from P1. For instance, in mice the fully processed form of P2 represents a slightly larger molecule than protamine P1. In humans, apes and Old World Monkeys two differently processed forms of protamine P2 could be found [4]. Another point is that P2 binds zinc ions. Experiments on intact sperm from various species were performed, and a coordination from one zinc atom per P2 molecule was found for human, mouse and hamster P2 protamines [58]. However, as long as the conserved histidine and cysteine residues are present, it seems like none of the different proposed zinc-finger models are consistent. The majority of the P2 sequences is needed to wrap around and coordinate the zinc ions, further, structures like these are not expected to bind to DNA sequences which are estimated to represent the P2 footprint [57].

Soon after their synthesis both protamines P1 and P2 get phosphorylated but when bound to DNA, most phosphate groups dissociate and the cysteine residues oxidize. Disulfide bridges are formed to link the protamines together [7]. Neighboring protamine molecules are cross-linked through this process, and thus a protection against removal or dissociation from DNA is provided until the sperm enters the egg [4]. The working group of Hutchinson et al. took a closer look on these bridges and proposed a torque force that reduces the packaging efficiency in mammalian sperm due to these inter-protamine disulfide bonds. Further, they also observed that the secondary P1 structure is needed for ensuring and supporting DNA condensation [59].

### 2.2. Molecular Function of Protamine

As already mentioned, packaging DNA in sperm, which implies protection of DNA against enzymatic degradation, and its compact condensation comprise the most important functions of protamine. A lot of excellent articles are discussing this matter [60,61]. The DNA binding capacity of P1 and P2 are differing. While P1 can bind 10–11 bp DNA, P2 protamines are able to bind about 15 bp and therefore a slightly larger DNA segment [62]. Dramatic nuclear DNA reorganization occurs during spermatogenesis. In mammalian sperm, a DNA condensation factor of ~40 can be seen [63], this condensation even reminds of crystalline packing levels [64]. This dense packaging helps protecting the DNA from UV radiation and damage [65,66]. 

The question of working mechanism then arises. During spermatogenesis, protamines act as nucleoproteins by replacing nuclear histones. Many protamine molecules bind nonspecifically to the DNA [57]. This binding leads to neutralization of the DNA phosphodiester backbone [4,67], consequently the condensation process begins and results in toroid DNA structures [68]. Sperm cells can have up to 50,000 toroids; each single toroid is able to store about 60 kb of DNA [69]. Several hypotheses can be found in literature about the toroid formation. A step-by-step folding process is proclaimed to be the dominant model. It is starting with a single loop of DNA and goes on loop-by-loop [68,70]. Very recently, Ukogu et al. took a closer look on the mechanism and observed that common models for DNA loop formation propose to be a one-step or rather an all-or-nothing model with a looped and an unlooped phase. They applied a Tethered Particle Motion (TPM) assay to evaluate the dynamic and real-time looping of DNA due to protamine and noticed the presence of reversible multiple folded states. Thus, they concluded that a multiple step process evoked by protamine, is bending DNA into a loop [71].

However, the DNA-protamine-complex stability is attributed to the combination of hydrogen bonds, electrostatic interactions and Van der Waals forces between the positively charged protamine and the negatively charged DNA phosphate groups. This binding mechanism leads to neutralization of the DNA phosphodiester backbone and further to fixed into place protamines due to the occurring network of disulfide bridges during epididymal transit. The male genome and the start of embryonic development is induced by this inactivation of the majority of spermatid genes. Furthermore, this aspect also ensures that the male genome in the sperm does not interact as a testicular cell when fertilizing the egg [4,72]. Protamine’s ability to bind DNA and other negatively charged biomolecules is recently used in various pharmaceutical fields. 

### 2.3. Protamine Derivatives 

A crucial aspect in medical applications is toxicity. It is worth mentioning that derivatization has influence on protamine’s efficacy as well as tolerance and toxicity. Therefore, the most common modifications are to form sulfate or chloride salts, reducing arginine molecules to decrease positive charges (low-molecular-weight protamine-LMWP) or to add attach molecules such as polyethylene glycol (PEG) [9,29,52,73]. Since 1969, protamine sulfate is approved for medical use in the USA and it represents the only protamine with a monography in the European Pharmacopoeia as well as in the USP. It consists of sulfates from basic peptides extracted from sperm of *Salmonidae* or *Culpeidae*. Nowadays, a recombinant production is also possible. The most common application field of protamine sulfate is surgery, where it is used as an antidote against heparin overdoses. However, protamine sulfate has much more properties, and researchers are using it e.g., as cell penetrating peptide (CPP) or as part of drug delivery systems like nanoparticles or liposomes [8]. In the year 1999, the working group of Yang discovered LMWP as a peptide fragment produced from native protamine (sulfate) by enzymatic digestion with thermolysine [13]. High output and rapid production of LMWP is enabled due to this method which also offers the advantage of being cost efficient and short manufacturing periods [74]. They published over 30 papers describing and evaluating the properties and applications of LMWP [9]. Further, they proposed less toxicity as well as lower immune response when applying LMWP as heparin antidote in comparison to the native protamine and very high efficacy when used as gene carrier in vitro [52]. 

## 3. Protamine in Various Pharmaceutical Fields

Protamine does not represent a completely new invention in pharmaceutical fields. So far, several protamine products have been available on the market for many years. Thus, it constitutes a well-established pharmaceutical ingredient [26]. To examine its different application fields chronologically, protamine was firstly used in therapy of diabetes mellitus. Combining protamine and insulin results in a prolonged effect of insulin which leads to lower blood glucose levels in patients [11]. Later, it was noticed that protamine can neutralize the anticoagulant effects of heparin and thus was applied as antidote in cardiac or vascular surgery to prevent postoperative bleeding events [15,75]. As one of the most remarkable findings, it is possible to use protamine as delivery system for biomolecules, such as CPPs for in vivo gene transport. The researchers mostly focus on protamine’s cell penetrating and nucleus targeting properties [8,27,29,76,77]. In addition, there are several working groups introducing protamine in different nanosized formulations to enhance cell penetration [24,78,79]. Another application field of great interest-especially in these difficult pandemic times-is the approach of using protamine in (mRNA) vaccines [80,81]. 

### 3.1. Protamine in Insulin Preparations

Applying insulin in the treatment of diabetes mellitus is a well-known form of therapy. When first introduced, protamine was used to prolong the action of insulin preparations. Thereby, protamine is combined with insulin to manufacture a protamine-zinc-insulin complex and neutral protamine Hagedorn insulin (NPH), respectively. First created in 1946, NPH insulin is an insoluble intermediate-acting insulin preparation which is applied once or twice a day [11]. The FDA approved NPH insulin for the control of diabetes mellitus type 1 as well as type 2. Currently, it is the most often used basal insulin and offers a sustained release of insulin over a prolonged period of time [82].

### 3.2. Protamine-Haemostatic Properties

At the beginning of the 20th century, it was proven that adequately dosed protamine-mostly given as protamine sulfate-reverses heparin’s anticoagulation effects. Inter alia, one important area of application field is heart surgery, especially cardiac surgery with cardiopulmonary bypass to treat bleeding events [82]. The ability to reverse anticoagulation of heparin is also utilized in the setting of dialysis, acute ischemic strokes and invasive vascular procedures [83]. Conventional injections (Protamine sulfate injection, USP, Fresenius Kabi or Protamin ME 5000 I.E./mL or Protamine chloride, MEDA Pharma GmbH & Co.KG, Stuttgart, Germany) are indicated for the treatment of heparin overdosage in general. The injection is applied intravenously, and it has a rapid onset of action, typically the neutralizing effect occurs within 5 min [84]. Again, the positively charged arginine groups are responsible for the antagonizing effect because they lead to electrostatic interactions between the highly acidic heparin and basic protamine. At a precursor ratio of 1:1 clearly visible, neutral protamine-heparin salt complexes occur within seconds. During the complexation, the original anti-thrombin-heparin complex dissociates which enables regular anti-thrombin activity again [15].

It has been noticed that the molecular weight of heparin is an important parameter for protamine’s neutralization efficacy. Smaller heparin molecules (low-molecular-weight-heparin) are more challenging to neutralize than larger molecules [85]. Binding to heparin is not the only haemostatic mechanism of protamine. There are also effects in relation with platelet functions as well as interference with coagulation factors and indicators of clot breakdown stimulation. Hecht et al. questioned adequate dosing and gave answers to the protamine conundrum [86]. The dosage of protamine is crucial for the success in reversing heparin induced anticoagulation. If protamine is administered in too-high doses, it promotes the anticoagulant effect of heparin and worsens the situation [15]. Despite that, several other emerging side effects are associated with protamine administration, like immunological and inflammatory alterations. Severe allergic reactions occur, including anaphylactic responses with low blood pressure, bradycardia and pulmonary vasoconstriction [87]. An increasing patient risk factor for anaphylaxis comprise diabetes mellitus treatment with protamine-containing insulin and allergic responses to fish proteins.

## 4. Protamine as Peptide-Based Drug Delivery System

The use of protamine also presents an attractive approach in the field of molecular biology and drug-delivery systems for biomolecules. Thereby, the cell-penetrating and nucleus-targeting properties of protamine are mainly into spotlight. 

### 4.1. Cell Penetrating Peptides (CPPs)

There are molecules like proteins and peptides which are used or developed to bypass the limitations of conventional therapeutics and deliver therapeutic macromolecules [17]. These conventional therapeutics are small molecules with low molecular weight that can modulate biochemical processes in order to treat, prevent or diagnose diseases. Classic examples are acetylsalicylic acid or diphenhydramine which have been playing a crucial role in shaping the world like it is today. Besides their important impact on today’s sophisticated health care system, their broad acceptance and easy handling for patient and pharmaceutical engineers pushed them in the position of one of biggest blockbusters in the history of the pharmaceutical industry. 

Unfortunately, besides their success, they have one big disadvantage. Typically, small molecules are mimicking biological substrates or allosterically target hydrophobic pockets of proteins. However, not all of these biological targets are druggable [88]. Therefore, the use of so-called cell penetrating peptides, which are referred to as not following the Lipinski rules of a regular drug molecule, represent promising and highly interesting alternatives or additions [17]. The attractiveness of CPPs lies in their targeting abilities—it is possible to reach specific molecules using biological pathways and consequently influence their effects and activities in a positive or negative way [89]. 

One of their biggest advantages is that they are capable to enter the cells in a noninvasive manner; thus, the integrity of the cellular membranes is not destroyed. Their way of penetrating the cells is considered as highly efficient and safe [22]. Additionally, CPPs show low cytotoxic effects and no immunological response [28]. Principally, CPPs comprise a maximum of 30 amino acids where most of them are basic amino acids like arginine. A consequential positive charge is also characteristic. Based on their individual properties and depending on their interaction with the therapeutic agent, a classification can be implemented. Our own working group [89] and several other authors presented detailed reviews on CPPs, their classification and internalization mechanisms [90,91,92]. Briefly, to distinguish the CPPs, two main classes regarding the binding strategies are mostly used. CPPs, capable of forming covalent conjugates with the cargo due to chemical cross-linking or cloning, represent the first group. As a result, a CPP fusion protein will be expressed. Examples from this class include transactivator of transcription (TAT) derivates or penetratin [93]. It seems apparent that the second class includes CPPs which bind their cargo noncovalently. Often, they have an amphipathic nature consisting of a hydrophobic and a hydrophilic moiety. By means of the CPP length and the interplay between the hydrophilic and the hydrophobic compounds this CPP class can be divided in three subtypes: the primary amphipathic, the secondary amphipathic or the non-amphipathic CPPs. More than 20 amino acids, which are sequentially arranged, determine the primary amphipathic peptides. Conversely, the secondary amphipathic CPPs mostly comprise less than 20 amino acids in their sequence. After interaction with the phospholipid membranes, they can take their α-helix or β-sheet conformation [90,94]. The third subtype constitutes the non-amphipathic peptides which are rather short and comprise a high content of positively charged amino acids like lysine and arginine [91]. Protamine belongs in this class of CPPs.

### 4.2. Game Changing Nanotechnology and Protamine’s Approach in this Novel Field

The first, but most likely unknown, use of nanotechnologies has been dated to the ancient Romans in the 4th century AD. The Lycurgus cup is exhibited in the British Museum and highlights one of the most outstanding applications of nanoparticles in ancient glass industry [95]. Bayda et al. published a detailed and very interesting review about the history of nanoscience and nanotechnology, manufacturing nanosized formulations as well as their successful story [96]. Nanotechnology represents one of the most promising techniques of the 21st century. Nanoscaled preparations like nanoparticles or liposomes incorporating CPPs are getting more and more popular because of their ability to deliver macromolecules as well as forming nanoplexes [97]. DNA as well as RNA nanotechnologies have become an interdisciplinary research field where researchers from pharmaceutical sciences, chemistry, physics, medicine and computer science are coming together to overcome obstacles and find solutions for future challenges [51,98,99,100].

**Figure 2:**
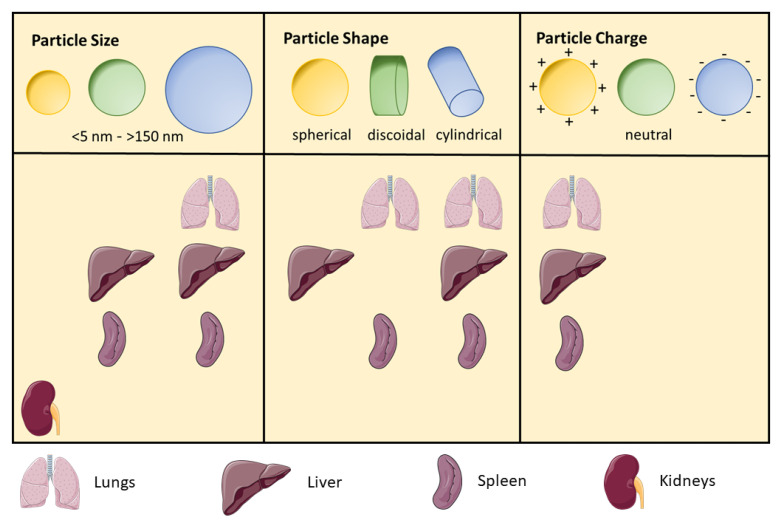
Impact of NP properties on their biodistribution in lungs, spleen, kidneys and liver. Especially NP size, shape and surface charge are dictating the biodistribution. Particles smaller than 5 nm are filtered by the kidneys. With increasing size (20–150 nm) higher amounts of NPs are detectable in liver and spleen. Even more NPs are entrapped in liver, spleen and lungs when the size is over 150 nm. It is said that these NPs are proven for long-lasting circulation [101,102]. Cylindrical shapes seem to be quite favorable because a lot of these NPs are distributed in lungs, liver and spleen but also discoidal forms exhibit high accumulation capacities [103]. Positive surface charges of NPs lead to a prioritized sequestration in lungs, liver and spleen. NPs with slightly negative or neutral surfaces show longer circulation times and lower accumulation in these organs [104]. Regarding the NP shape and surface charge data, it is important to mention that the size of the discussed NPs is said to range from 20 to 150 nm [101].

With respect to nanoparticles, several physicochemical parameters are essential for predicting their application potential in vitro and in vivo and for their use in future pharmaceutical strategies. In Figure 2, an overview on how physicochemical properties can influence the biodistribution in several organs is given. 

According protamine nanoparticles, each formulation needs its own optimized mass ratio of the oligonucleotide (ODN) and protamine, which must be found experimentally. This is because the concentration is a crucial aspect concerning particle size, particle size distribution, zeta potential, drug load, binding strength and transfection as well as drug release efficiency [101,105,106]. When it comes to biological barriers and strategies or rather nanoparticle designs to overcome them, particle size plays a crucial role. It is a parameter which can easily be influenced from the manufacturing point of view and determines the uptake preferences of the organs. Larger particles (>150 nm) are known to preferentially enter lungs, liver and spleen but not the kidneys. But a nanoparticle size <5 nm should help to achieve high accumulation in kidneys [102]. Additionally, it is possible to determine discrete cut-off size ranges which are impacting circulation half-life, extravasation through leaky vasculature and specific cellular uptake [107]. Nevertheless, nanoparticle shape is another critical feature. According to “the form follows the function” this property influences the biochemical behavior heavily [101]. The architecture of the nanoparticles is affecting hemorheological dynamics as well as-again-cellular uptake in different organs and thus in vivo circulation fate. Spherical shapes (<45°) show faster internalization than nanoparticles with curvatures > 45° [108]. The third important parameter in overcoming biological barriers is the surface characteristic. Surface charge as well as hydrophobicity represent designable parameters too and lead to selective enhancement in accumulation at specific sides of interest. It is said that neutral or negative surface charge results in longer circulation half-lives, and positive charge leads to a higher rate of nonspecific uptake in the majority of cells [105,109]. When thinking of in vivo fate of nanoparticles, deformability and biodegradability are also to be considered. It has been shown that nanoparticle stiffness impacts biodistribution as well as circulation. This effect can be influenced by the degree of crosslinking in the nanoparticle [110]. Further, it is postulated that deformability might be an influencing parameter when it comes to the nanoparticle transport efficacy through small capillaries like in the lung [111]. Nanoparticle stability plays an important role in kinetics. Given that fact, it can be said that biodegradation is a major point in nanoparticle engineering [112].

Finally, these mentioned parameters also have an impact on cytotoxicity. Just to repeat the main influencing factors, they are nanoparticle size, shape, composition, surface charge and surface hydrophobicity [105]. The correlation between cytotoxic effects and nanoparticle size demonstrated that the smaller the nanoparticles the higher the cytotoxicity [113,114,115]. Moreover, spherical shapes work more compatible in cells than, e.g., fiber-shaped nanoparticles [116]. Regarding surface characteristics, it is said that hydrophobicity is often connected to surface charge. Nanoparticles with charged and hydrophobic surfaces, interestingly, show higher cytotoxic potentials than nanoparticles without hydrophobic properties. These effects were e.g., demonstrated with oleic acid-coated nickel ferrite and stearic acid-coated TiO_2_ particles [117,118].

Nanosized delivery systems for small biomolecules like mRNAs, siRNAs or microRNAs have attracted a good deal of attention recently. Especially due to the actual Covid-19 situation, the discussion about pharmaceuticals incorporating different sorts of RNA is gaining more and more momentum. Therefore, our working group puts great effort into the improvement of biomolecule delivery systems. In the early 2000s, our research group invented special solid nanoparticles consisting basically of antisense ODN and protamine. These formed nanoparticles are so-called “proticles”. The condensation occurs due to the electrostatic interaction between the negatively charged ODN and the positively charged protamine and results in nanoparticles in a size range of 100–200 nm [76,119]. Two main disadvantages have been noticed: on the one hand, secondary aggregation of the proticles, which is highly dependent on their concentration, may occur in presence of salt, and on the other hand, poor intracellular dissociation of the two components is observed which leads to low cellular efficacy [51,77]. To resolve these issues, modifications on the binary system have to be done.

#### 4.2.1. Manufacturing Protamine-Based Nanoparticles

**Figure 3:**
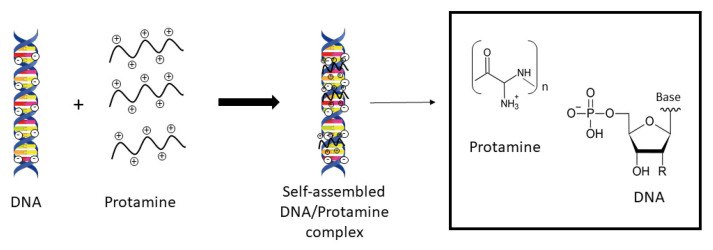
Scheme of self-assembling process between the positively charged Protamine and DNA as negatively charged ODN component. Electrostatic interactions provoked by Protamine’s cationic amino acid groups and the negative phosphate backbone of the ODN result in a self-assembled ODN/Protamine complex.

Top-down and bottom-up manufacturing methods are proposed to be the two approaches to achieve nanostructures. They differ in degrees of their quality, production speed and manufacturing costs. During the top-down processes, bulk is crushed or shred into nanosized structures. On the other hand, nanostructures are pieced together from smaller systems when using bottom-up methods. Atom-by-atom or molecule-by-molecule can be linked together by physical and chemical methods. Controlled manipulation of self-assembly properties of the atoms or molecules is applied [120]. In 2006, Paul Rothemund described the “scaffolded DNA origami” by investigating the characteristics of self-assembled DNA nanostructures in the so-called “one-pot” reactions [121]. A scheme of the self-assembling process by means of DNA is given in Figure 3.

There are two important points when it comes to the self-assembly properties. First of all, positional assembly is the only technique which allows single atoms or molecules to position themselves freely, one-by-one, and secondly, the manufacturing itself is quick and easy, which makes it cost-efficient [96]. Junghans et al. demonstrated that the mixing of aqueous protamine and ODN solutions in a well-defined mass ratio provoke immediate self-assembling. A discoloration from transparent to opaque indicates the presence of nanoparticles, verified by investigating the particle size distribution by light scattering techniques and imaging using electron microscopy. Further, it was shown that particle formation is possible for modified phosphodiester as well as phosphorothioate (PTO) ODNs [76]. However, a minimum chain length of nine nucleotides per ODN is required for successful particle preparation [119]. Scheicher et al. scrutinized the self-assembly manufacturing process with Proticles consisting of protamine, ODN and secretoneurin. They mixed ODN with secretoneurin before protamine addition and compared the classic preparation process, in which the protamine and ODN–secretoneurin solutions were combined in one working step, to a nanoparticle formation by protamine titration. Protamine solutions were divided into seven equal aliquots and added separately to the ODN solution. The data imply that the nanoparticle manufacture by titration facilitates the modification of particle size, which is most probably connected to the second titration step. Only the applied mass ratio, but not the manufacturing method, influenced the drug loading [27]. 

Petschacher et al. focused on the upscaling process of self-assembled nanoparticles consisting of a thiomer and protamine in a microreactor. They noticed that the mixing process to a great extent determines the particle size and the particle size distribution. Therefore, mixing is a crucial parameter to consider. It is worth mentioning that their unprecedented approach of the passive microreactor for producing biodegradable thiomer–protamine nanoparticles by electrostatic self-assembly succeeded [122]. 

#### 4.2.2. Functionalizing Proticles

Nanoparticle engineering and functionalization is a challenging task and requires a lot of experience as well as creativity. Chemical ODN modifications like PTOs are helpful in terms of stability issues. They are widely used to prevent enzymatic degradation and enhance efficacy [51,79]. The application of protamine sulfate instead of protamine free base represents another modification possibility and results in a drastic particle size reduction. Unfortunately, no improvement in cellular uptake or intracellular drug release could be observed [78]. Supplementation is another enhancing strategy. In this case, the conventional binary proticles were expanded to a ternary system by incorporating a third component. Hereafter, we describe some selected approaches.

An older, but effective, method is the use of human serum albumin (HSA). Pharmaceutical nano- and microsciences are common application fields of HSA because of its beneficial properties in particle formation and intracellular efficacy as well as its nontoxic characteristics. Due to its negative charge, it can bind positively charged biomolecules like protamine. Thus, it is proposed that HSA serves as a transporter of a variety of different ligands [123,124,125,126,127]. Albumin supplemented proticles were prepared by combining modified or unmodified ODNs with aqueous mixtures of protamine and HSA. In this way successful binding to protamine as well as incorporation in the nanoparticles could be assured when mixed with the ODN solutions. Ternary proticles comprising HAS-supplements demonstrate higher stability towards nucleases and slower agglomeration tendency. Moreover, they are able to achieve sufficient stability in salt solutions in comparison to the binary proticles. Superior cellular uptake and intracellular ODN distribution was also noticed. Especially HSA-PTO proticles have proven to be advantageous. To a large extent these alterations are attributed to the conformational change of HSA at endosomal pH [128]. HSA shows fusogenic activities under acidic conditions, which may result in endosomal destabilization and further improve intracellular drug delivery [51,77]. As already mentioned, proticles without HSA show aggregation tendencies in salt solutions which correlate with instabilities.

**Figure 4:**
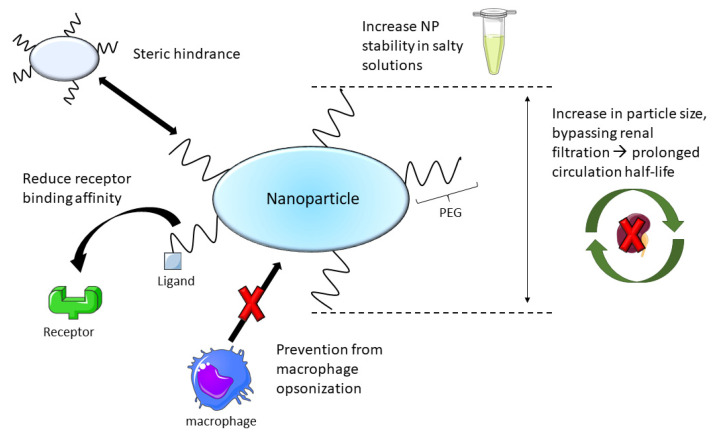
Schematic depiction of the impact of PEGylation on NPs. PEGylation is a common strategy to modify and further functionalize NPs. It was shown that the use of PEG increased NP size and therefore prolonged the circulation half-life by evading renal filtration [29,137]. Moreover, it led to a reduction in receptor binding affinity [133], provokes steric hindrance [131], increased the NP stability in salty environment [140] and it prevented the NPs from opsonization by macrophages [138].

Next to albumin, PEGylation offers another well proven option increasing nanoparticle stability. But PEG is not just known for its stabilizing effects. Figure 4 highlights the impact of PEGylation on NPs. Depending on the chain length and molecular weight, the pharmacodynamics, pharmacokinetics as well as targeting efficacy can be regulated [129,130,131]. Further, important parameters in formulation development are the PEG ratio and the mode of attachment. Many effects can be found in literature, such as increasing solvent viscosity which is correlated with a retardation in particle growth [132]. Steric hindrance [129] to reduce receptor binding affinity [133] can be provoked as well as the (positive) surface charge of the nanoparticles preserved or shielded. These effects may influence cellular uptake and/or endosomal escape [134,135]. PEG implementation also helps evading renal filtrations which is resulting in prolonged circulation half-life [136,137,138]. Another remarkable property of PEG is making nanoparticles “invisible for the immune system” and thus preventing them from opsonization by macrophages [136,139].

By the PEGylation of proticles, many of these effects can be adopted for functionalization. Lochmann et al. administered PEG 20,000 in order to use it as stabilizer for Proticles in salt solutions. In this work, the binary proticles were produced and afterwards incubated in various PEG-20,000 solutions, which represents a kind of coating process. They succeeded in their goal in increasing their stability in cell medium but because of physiological incompatibilities, further developments are required [140]. In accordance to PEGylated proticles, Fresacher et al. applied another functionalization method in which protamine was derivatized with diethylenetriaminepentaacetic acid (DTPA) and PEGylated with PEG-2000 before nanoparticle formation. A comparison of PEGylated and non-PEGylated Proticles with respect to their in vitro stability and in vivo biodistribution was performed. For this reason, the Proticles were radiolabeled with ^111^In^3+^. Nanoparticle stability in serum and PBS was determined, as well as biodistribution in rats. Interestingly, the stability decreased due to PEGylation but on the other hand prolonged half-life and an increased accumulation of the PEGylated proticles, particularly in liver and spleen, was observed. Renal excretion route has been investigated as the major elimination pathway [29]. To conclude, PEGylation seems to be an efficient tool to improve the properties of proticles but still needs optimization to gain a key position in proticle engineering. 

An advanced form of nanoparticles are solid lipid nanoparticles (SLNs) including protamine. In general, SLNs represent effective carrier systems in gene therapy. They can overcome main biological barriers and show important advantages like their composition of well tolerated physiological lipids and their easy large-scale manufacture. Further, sterilization and lyophilization of SLNs are possible which lead to good storage stability [141,142,143]. Basically, SLNs are consisting of solid lipid cores which are surrounded by a layer of tensides in aqueous dispersions. Mostly positively charged surfactants are applied in order to obtain cationic SLNs, binding nucleic acids or ODNs due to electrostatic forces [144]. However, sometimes anionic SLNs are produced with the ability to induce transfection. But in this case the nucleic acid has to be previously bound to a cationic ingredient like protamine [25,145]. A crucial aspect for successful drug delivery includes the necessity of nucleic acid condensation, ensuring sufficient transfection efficacy [144]. An equilibrium of condensation, protection and ODN release is mandatory to achieve good transfection levels [141]. He and coworkers prepared ternary cationic SLNs incorporating protamine by manufacturing the classic binary proticles in first row and adding the protamine/DNA nanoparticles to a cationic SLN dispersion afterwards. The objective of their research was to design an even more effective drug delivery system (DDS) for DNA than the original proticles. Their investigations exhibited that due to SLN formation an enhanced entry into HEK293 cells occurred and protamine protected the DNA from enzymatic degradation [25]. In another study, researchers engineered SLNs with attached dextran–protamine DNA complexes on their surface. Therefore, the initial dextran-protamine–DNA complex was formed and afterwards added to the SLN suspension. Due to interactions between the free negative DNA charges and the positive charges of SLNs a stable DDS could be formed. Depending on the cell model, a higher transfection capacity due to dextran and protamine could be found. Moreover, their vector system was able to induce marker expression in liver, spleen and lungs of BALB/c mice, which could be tracked for at least 7 days. In comparison, the application of free DNA did not lead to any expressing activities [24].

Anionic solid lipid nanoparticles incorporating protamine and DNA were prepared by forming the binary protamine–DNA complex and sequential addition of anionic lipid nanoparticle dispersion. These lipid nanoparticles were basically consisting of different ratios of monostearin and oleic acid. Once more it was highlighted that cell treatment with SLNs supplemented with protamine and DNA show high cell viability in various cell types and a significant increase in transfection efficacy due to functionalization of the binary proticle system [145]. 

In addition to this aspect, Junghans et al. have shown the loading of proticles into liposomes. The combination of proticles with cationic lipids improved the ODN loading capacity and lowered the cytotoxicity of the liposomes. They also noticed an increased sequence specific antisense effect throughout their investigation [53]. With respect to all mentioned studies and formulations, one point is clear: the success of the delivery system and its toxicity always depends on the ratio between protamine, the ODN and the supplements. 

Despite several already discussed points, like protection and sufficient drug release of the active pharmaceutical ingredient (API), targeting is another crucial parameter when inventing a potent carrier system. Therefore, targeting strategies have been developed over the last decades. Different methods like coating or co-assembling of targeting sequences have been established. Proticles were successfully loaded with vasoactive intestinal peptide (VIP) in 2008. A depot effect due to proticle assembling and prolonged pulmonary vasodilator activities could be found [146]. Further, it was concluded that the combination of high VIP loading capacities and the extended effect represent a promising approach for sustained peptide-based DDSs. Two years later, proticles were again loaded with VIP to target vasoactive intestinal peptide receptor (VPAC) overexpressing tumor cells, published by Ortner et al. [147]. The results demonstrated an accumulation of the VIP loaded nanoparticles at the surface of VPAC expressing cells followed by the internalization of physiological active VIP. 

Another peptide for functionalizing proticles is apolipoprotein A-1 (Apo A-1). Proticles were coated with Apo A-1 to enhance receptor mediated endocytosis by imitating lipoprotein particles [148]. Kratzer et al. managed to overcome the blood–brain barrier utilizing the same coating. The comparison of coated and uncoated nanoparticles showed a remarkable improvement in transcytosis through brain capillary endothelial cells [149]. Deeper regions of the brain could be targeted by coating proticles with Apo A-1. 

In the diagnosis field, proticles with targeting supplements were established. Almer et al. linked signal-emitting molecules to proticles in order to detect atherosclerotic plaques. Adiponectin-coated nanoparticles demonstrated better noninvasive imaging properties. After some years, the same group published an improved IL-10 mediated targeting strategy. They noticed differences in distribution between proticles and targeted liposomes in mice ex vivo [150]. Secretoneurin was incorporated into proticles by co-assembling, as mentioned above. The aim of this research was to develop a successful delivery system for secretoneurin and provide a novel therapeutic option in the treatment of, e.g., peripheral arterial diseases by applying a new nanoparticle manufacturing method. In an in vivo biodistribution study, they demonstrated a retarded distribution of secretoneurin after secretoneurin-proticle injection. This innovative nanoparticle production method also offers new possibilities for proticle engineering and handling with respect to stability and storing [27]. Very recently, researchers equipped protamine-based NPs with an aptamer to selectively target lymphoma cells to treat cancer. They fabricated proticles consisting of protamine, an oncogene-specific small interfering ribonucleic acid (siRNA) and an RNA-based CD30 aptamer. With this approach it was possible to achieve cell-selective chemotherapy delivery and oncogene-specific gene therapy at once. It was demonstrated that by means of this innovative idea, the NPs effectively killed the lymphoma cells and therefore they demonstrated their high potential for precocious therapy forms [151]. 

#### 4.2.3. Immunogenic Properties of Proticles 

Proticles are known to possess immune-modulating properties. This effect was first evaluated by applying CpG-oligonucleotides [44]. It was demonstrated in a very impressive way that proticles without immunogenic CpG-control-ODNs had no immunogenic response [46]. In the next section, we offer a detailed look on the potential and use of protamine as well as proticles in the field of vaccines.

## 5. Protamine and New Vaccine Technologies

At the start of a new decade, humankind was faced with a virus outbreak that reached the pandemic scale soon after it was discovered. This year-long fight with a nanosized “enemy” seems to have pushed forward a question of immense importance: where do we stand today in terms of vaccine development? Furthermore, are we prepared for a fast response when the world is in chaos? 

There is no doubt that vaccine development is one of humankind’s most important endeavors. Its impact on the relationship between infectious diseases and the human race can be seen in the eradication of smallpox and the restriction of diseases such as measles, polio, diphtheria and tetanus. Nonetheless, changes in the climate, population density, age distribution and traveling habits made easy the emergence and spreading of pathogens, new as well as old [152]. This highly dynamic modern way of life presented no difficulties in predicting a pandemic outbreak, such as the COVID-19 pandemic. The rapid spread of this severe infection brought to light the need of global alertness in response to a pandemic, which involves the rapid development and worldwide distribution of a vaccine that can potentially be directed towards an unknown pathogen. 

The conventional methods of vaccine production usually rely on the use of whole live, attenuated and inactivated pathogen or protein subunits. Yet, these well-established methods may not be suitable in outbreak situations. Live attenuated viruses always pose the risk of reversion into a highly pathogenic form. On the other hand, vaccines based on inactivated viruses and protein subunits may not be sufficiently immunogenic. In addition, producibility of the classic vaccines during an outbreak poses an issue as well, since they do require whole pathogen cultivation and propagation [152,153]. 

Having this in mind, we become aware of the great need for novel vaccine technologies, that would offer some advantages over the conventional ones, especially in the case of rapidly emerging viral diseases. Ideally, the vaccine platform in pandemic settings could be produced rapidly and in big quantities in order to satisfy global needs. A great hurdle in this case is cold chain storage, which makes transportation of vaccines to developing countries difficult. Thus, the design of a scalable and temperature stable vaccine is an ongoing challenge. 

Moving from the historical paradigm on which vaccine development has been based —Louis Pasteur’s ‘three Is’, isolate, inactivate and inject—vaccine development today is based on rational design. What this means is that the better understanding of immunology, pathology and microbiology is essential in the development of safe vaccines. The better understanding of molecular mechanisms that take place in pathogen–host interactions as well as the mechanisms of the immune system, aids in the design of more selective vaccines. These include vaccines based on virus-like particles as well as nucleic acid-based systems that offer increased robustness in antigen production, lower production costs and higher production rates. Furthermore, with the development of a suitable delivery system, targeted delivery of the antigenic material can be achieved, and the release profile can be controlled [154]. 

In this part of the review, we will focus on the key components of the immune system, novel vaccine technologies and, most importantly, methods for their delivery. When it comes to delivery systems, we will put our attention on nanoparticulate platforms, especially nanosystems composed of cell-penetrating peptides. Protamine, as a highly basic, positively charged cell-penetrating peptide, is the peptide of our focus.

### 5.1. Key Components of the Immune System

The immune system can be described as the protective component of our organism during infectious disease. This would be the traditional view or definition for immunity. Looking back at evolution, it seems that the immune system evolved because it provided host protection from pathogens, thus, it provided a survival advantage. However, pathogens are also selected to overcome the host resistance, which means that there is a well-established co-evolutionary dynamic. As much as this model stands correct still, today we are aware of the multiple functions the immune system has, one of it being the response during sterile inflammation and maintenance of tissue homeostasis [155]. The role of immunity in such complex processes implies that the immune system itself is an intricate network composed of numerous regulatory pathways, involving different cellular components as well as molecular counterparts. 

The immune system is made up of a plethora of cells, which can reside in specific parts of the body (such as the skin, respiratory, gastrointestinal and genital tracts), or they can circulate through the body scanning for invading pathogens [35]. These cells can be roughly grouped into two parts, that are viewed as the two main components of the immune system-the innate immunity, and the adaptive immunity. Nevertheless, these two cannot be regarded as separate, because there is always a form of communication between them. 

Innate immune cells are regarded as the ones responsible for a quick respond. Part of the “first responders” are polymorphonuclear cells (neutrophils, basophils and eosinophils), mast cells, macrophages and dendritic cells. While all of the cells mentioned have a specific mechanism of action when triggered by pathogens, worthy of attention are the macrophages and dendritic cells, also known as antigen presenting cells (APCs). These two groups of cells are capable of internalizing and destroying microbes through phagocytosis and then activating the cells of the adaptive immune system [32]. Pathogens are recognized by their conserved microbial products, called pathogen-associated molecular patterns (PAMPs). Dendritic cells and macrophages are activated by the interaction of PAMPs with so-called pattern recognition receptors (PRRs), such as the membrane-bound Toll-like receptors (TLRs) [156]. The interaction initiates a signaling cascade that ultimately results in generating pathogen peptide fragments by proteasomal degradation in the immune cells. These antigens are then presented on their surface, on receptors called major compatibility complex I or II (MHC I and MHC II). MHC I and MHC II are important for antigen presentation to and activation of naïve T-cells.

Another very important part of the innate immunity is the complement system, which represents the soluble or humoral part in the innate immune system. The complement is considered a cascade, composed of soluble proteins, membrane expressed receptors and regulators. There are three pathways of complement activation: the classical pathway (activated by immune complexes and apoptotic cells), alternative pathway and lectin pathway. Each of these involves a specific signaling cascade that will ultimately result in the activation of complement proteins. When activated, complement components tend to opsonize (or mark) pathogens in order to facilitate phagocytosis and help with the recruitment of phagocytic cells. The complement plays a central role in the modulation of T and B-cell responses, and after the generation of antigen-specific antibodies, it contributes to the clearance of immune complexes and pathogens [157]. 

The adaptive immunity is the one responsible for long-term immunological memory and it is the part of the immune system that needs longer time for activation and development. It is composed of two major components: T and B-cells. T-cells are generally classified in two groups, based on the surface receptor they express, CD4 or CD8. The key event for activating T-cells is the antigen presentation by APCs to a T-cell via the MHC I or MHC II pathway. When a T-cell receives a signal from APCs, it starts proliferating and producing antigen-specific T-cell clones [36]. CD8+ T-cells, also known as cytotoxic T-cells, are activated by the MHC I path, while the CD4+ T-cells, known as helper cells, are activated by the MHC II path. The cytotoxic T-cells, once activated, secrete cytotoxic granules and perforin that penetrate the target pathogen, thus killing the pathogen. CD4+ T-cells are referred to as helper cells, because they contribute to the cytokine response, that drives the immune response to either cell mediated immunity (by activation of macrophages and CD8+ cells) or humoral immunity mediated by B-cells. B-cells, on the other hand, circulate in the blood and lymph and provide surveillance for signs of infection. When activated, B-cells start producing and releasing antibodies that can bind to the target protein (antigen) and neutralize it. At this point, B-cells are known as plasma cells [32,35]. Although a large part of T- and B-lymphocytes will be activated and fight the infectious agent, a group of them continues to dwell within lymph node compartments, forming immunological memory or memory cells. This means that in the case of reinfection with the same or slightly different pathogen, these memory cells will react much quicker than naïve lymphocytes.

#### Immune Response after Vaccination

The main principle of vaccination is the induction of a protective immune response by mimicking the natural infection caused by a pathogen (bacteria, virus etc.). The difference, however, between a natural infection and the reaction caused by a vaccine is that vaccination eliminates the risk of acquiring a disease with all of its potential complications [158]. Therefore, a vaccine contains one or several antigens that resemble a microorganism, that are able to stimulate the body’s immune system. 

The innate and the adaptive system work in unison in order to elicit an immune response, after a vaccine has been applied. The onset of activities is driven by antigen presenting cells—notably, dendritic cells, which recognize the PAMPs introduced with the vaccine. As mentioned earlier, an important family of PRRs that helps in the recognition of PAMPs is the Toll-like receptor family (TLRs). TLRs are membrane-bound glycoproteins, found on the cellular membrane or located intracellularly, as part of the endosomal membrane [31]. Membrane-bound TLRs are capable of interacting with ligands (or commonly known as epitopes) present on the surface of the antigen itself. However, the endosome-located TLRs require their ligands, which mostly are viral nucleic acids, to be internalized and digested in order for signaling to occur. Following the recognition of PAMPs, dendritic cells are trafficked to the lymph nodes, where they come in contact with naïve CD4+ and CD8+ T cells. They are stimulated to proliferate and further activate B-cells to produce antigen-specific antibodies. Most antigens used as vaccines can stimulate both T and B-cell production, however, the nature of the vaccine can influence the nature of the effector cells that are predominantly activated. This mostly depends on the nature of the antigen, administration route, quality of antigen presentation, vaccine adjuvants etc. [159]. 

Nevertheless, novel vaccine technologies struggle with a recurring problem, and that is-lower immunogenicity than the conventional live attenuated or inactivated pathogens. This is probably due to the fact that conventional vaccines have a multitude of antigen structures that can be recognized as epitopes and can be opsonized, while the novel highly purified and defined antigens might lose some of their immunogenicity during the purification processes. The solution to this problem comes in the form of “adjuvants”, i.e., tools that can help with the activation of the immune system. The most commonly used adjuvants are aluminum salts and oil-in-water emulsions [160]. Other novel adjuvants include liposomes, polymers, peptides, inorganic particles and immune-stimulating complexes, which also might act as carriers for the vaccines [161]. In general, these “helpers” are known to elicit strong cellular and humoral responses. Furthermore, adjuvants are known to interact with PRRs, especially TLRs, in a way that PAMPs would. This is helpful in activating T-cell mediated response, if we have in mind the fact that some of these molecular patterns might be lost during the purification process of the antigen. The topic of vaccine adjuvants that also function as their carrier systems, will be reviewed in more details in the following chapters.

### 5.2. Novel Vaccine Technologies

**Figure 5:**
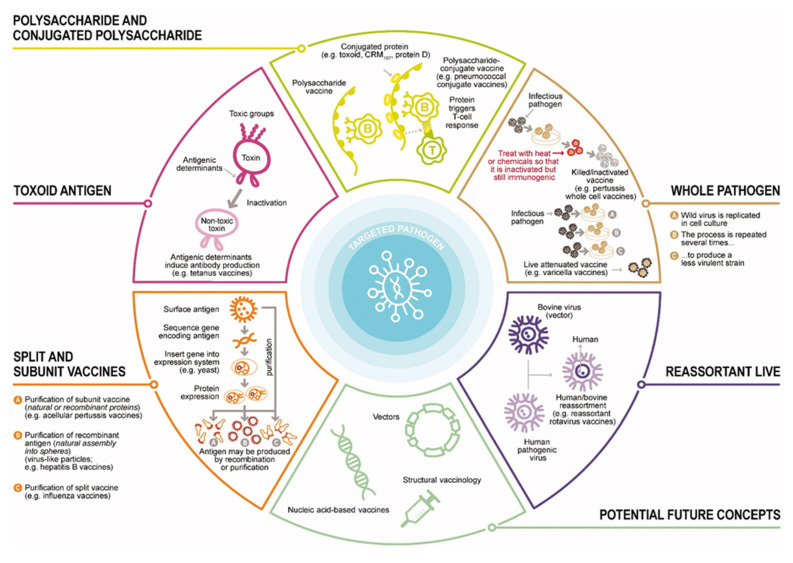
Types of vaccines being developed. Vaccines can contain live, whole pathogens, inactivated pathogens, toxoids, and parts of the pathogen. Novel concepts include vectors as delivery systems, and nucleic acid-based vaccines. Reprinted from [158]. CC-BY 4.0 (https://creativecommons.org/licenses/by/4.0/), accessed on 21 January 2021.

Vaccines represent one of humankind’s most significant advancement in public health. Thanks to the development of vaccines and successful vaccination programs, morbidity and mortality are prevented and reduced in millions of people each year. As mentioned earlier, traditional vaccine development relies on the use of whole organisms, either live attenuated or inactivated. No matter how successful these vaccines have proved to be in the treatment and eradication of diseases, they still carry some disadvantages. Their production process is lengthy and expensive, it requires culturing of the pathogen, and there is always the risk associated with their safety. The safety issues namely include the possibility of reversion of the pathogen to its full pathogenic form, possible mutations or incomplete inactivation of the antigens in the production process. This is the reason why novel technologies are leaning towards the production of cost-effective, and safe highly purified vaccines, that would be more specific in activating the immune system. Included here are recombinant proteins, known as subunit vaccines, as well as nucleic acids. The problem of these vaccines, as mentioned before, is the lower immunogenicity compared to conventional whole organisms. A solution for increasing the immunogenicity is the use of adjuvants-smart tools that help boosting the immune system. Another field of extreme interest today is the application of nanotechnology, which would allow particulate systems in the nano range to be used as carries for the antigen of interest. Furthermore, these types of nanoparticles can be used as adjuvants. As such, besides acting as the carrier system for the antigen, they could also play an immunostimulatory role [33]. Figure 5 gives a brief overview of the types if vaccines we have today. 

In the following text, we will give a brief overview of the history of vaccines, as it is of great importance for understating the deduction method by which we came to the simpler vaccines we have today. In addition, adjuvants and the use of nanotechnology for vaccine delivery and immune stimulation will be discussed.

#### A Brief History of Vaccinology

The saying goes that only those who have understood the beginning of things can also understand the present. With the explosion of new strategies for vaccine development, and more than a 200-year history of vaccination, it is more than useful to contemplate the past. The early history of vaccines can be reduced to empirical discovery, without any real immunologic rationale, as something similar to black magic. The ways of discovery have shifted far from their origin today, and strategies based on genetic engineering, systems and structural biology aid in a great way in achieving a protective immune response [162]. 

At the beginning, there was smallpox. The first documented attempts to prevent smallpox infection come from Middle Eastern and Asian cultures, where the pustules from patients were taken and dried, and then inhaled or scratched onto the surface of another patient’s skin. The concept of inoculation of the infective material, called variolation, was introduced to the Western world in 1718, by Lady Mary Wortley Montagu, wife of the British ambassador in Turkey. After getting familiar with this practice in Turkish communities who escaped smallpox, she had her children variolated to prevent them from becoming infected with the disease [163]. Subsequently, the practice of variolation or inoculation became common in the United Kingdom. 

The concept of vaccination was introduced to the world by Edward Jenner at the end of the 18th century. After observing that patients who had contracted cowpox were resistant to variolation, or natural smallpox infection, he postulated that their cowpox “immunity” is very long lasting. He had the idea that by inoculating people with the material contained in cowpox pustules, they would be protected against a future smallpox infection. His first ever vaccine trial was performed in an 8-year-old boy, by inoculating matter taken from cowpox pustules from a milkmaid in small incisions in his arm. After being variolated with smallpox, the boy showed no symptoms of the disease. Although vaccination was a cause for many concerns, as it was not regarded as safe as variolation, it became the standard procedure for smallpox prophylaxis after the ban on variolation in 1840 [164].

The following important point in vaccine history is the concept of attenuation. This was brought forward by Louis Pasteur, while studying and working on chicken cholera. Pasteur was successful in culturing the causative agent of cholera in suboptimal conditions. He later observed that these cultures had lost their virulence when inoculated in chickens, but they were still immunogenic and able to induce protection against the disease. This was noticed after challenging the animals with the lethal strain. Pasteur termed this procedure vaccination. After having numerous successful vaccination procedures in animals, he had the first success in human vaccination. This followed the discovery of transmission of rabies via dog saliva. Pasteur was able to isolate the infective agent, attenuate it by passaging from dogs to monkeys, and finally, vaccinate a boy who had been bitten by a rabid dog with a low chance of survival. The treatment was successful, and the boy survived. Luis Pasteur’s concept of vaccination resulted in rabies mortality drop to 0.5% [162,165]. 

A breakthrough in the mid-twentieth century launched what is known as the golden age in vaccinology. This period was marked by the development and improvement of techniques for maintenance of animal cell cultures. Since viruses are intracellular parasites that need a host in order to grow and reproduce, it was of great importance that effective cell and tissue cultures are developed. By this time, scientists were able to propagate viruses even in human tissues [166,167]. This success was followed by the development of two different types of polio vaccines, an inactivated and a live vaccine [168,169]. At the same time, it was demonstrated that immunoglobulins, or antibodies, are the ones responsible for the immune protection against the three types of polio virus. The development of three other attenuated-virus vaccines also took part in the so-called golden age. These were vaccines against childhood diseases: measles, mumps and rubella. In the second half of the twentieth century they were combined into a single vaccine, one we know as the measles, mumps and rubella vaccine (MMR) [170,171].

The last phase in vaccine development is ongoing, and this is the era of genetic engineering. The revolution in biology allowed the use of bacteria, yeast and animal cells as substrates for the production of immunogenic proteins. By using recombinant DNA technology, antigens from otherwise unculturable or highly pathogenic infective agents can be produced in high amounts in vitro. These are the so-called subunit vaccines, and they include purified proteins (virus-like particles and toxoids), polysaccharides, protein-polysaccharide conjugates, glycolipids or lipoproteins. Today, there are subunit vaccine candidates for a plethora of diseases, such as HIV and malaria [172,173,174]. However, as mentioned earlier, the subunit vaccines lack the immunogenicity, that whole organism vaccines have, due to the fact that they only contain one copy of the antigen. One approach that aids in this problem is the development and use of adjuvants, a topic that will be tackled in the following chapter. 

### 5.3. Adjuvants—Components to Boost the Immune Response

The use of highly purified antigens as vaccines commonly results in the induction of a modest immune response and thus, requires the use of multiple vaccine doses in order for sufficient antibody response to be elicited [175]. Therefore, the use of an adjuvant would facilitate the use of smaller doses, the induction of immunity following immunization protocols based on fewer doses of the vaccine, and, last but not least, the adjuvant would increase the stability of the vaccine. This is of great importance, because it means that the vaccines would be less susceptible to degradation during storage [161]. 

An adjuvant is commonly defined as a compound which is added to a vaccine in order to enhance the immune response, and the definition of an adjuvant usually comes from what it does and not by its nature. For simplification purposes, adjuvants are grouped in two groups: immune potentiators and delivery systems [176]. Immune potentiators work by directly activating the immune system. They can be generated from parts of a pathogen or can be synthetically produced-like unmethylated CpG DNA (single stranded DNA molecules) or lipopolysaccharide (LPS) coming from bacteria or double-stranded RNA molecules [177]. Most of the immune system potentiators are ligands for Toll-like receptors (TLRs), NOD-like receptors (NLRs), retinoic acid-inducible gene I (RIG-I)-like receptors (RLRs) etc. Delivery systems, on the other hand, act by promoting the uptake of antigens in immune cells. Alum, emulsions as well as particulate systems fall into this category [178,179]. Nowadays, however, the approach is more focused on combining immune potentiators and delivery systems. This allows the safe delivery of the antigen to the immune cells of interest, like dendritic cells, and increase the antigen presentation in order to facilitate the activation of the adaptive immunity by stimulating the innate immunity [180,181]. Nevertheless, only a few adjuvants have been licensed for human use, and, even for them, the exact mechanism of action is still not elucidated. These include aluminum salts, oil-in-water emulsions (MF59, AS03 and AF03), virus-like particles and liposomes [182]. 

#### 5.3.1. Mechanism of Action

Adjuvants are able to act by a combination of mechanisms, such as depot formation, recruitment of immune cells, enhancement of antigen uptake and antigen presentation, induction of cytokines and chemokines.

##### Formation of Depot at the Site of Injection

The formation of depots at the site of injection might be the oldest suggested mechanism of action of adjuvants. Antigens can be adsorbed on the surface of the adjuvant, or “trapped” inside of it, so forming a depot would allow a sustained release profile of the antigen, which would mean that the organism would be exposed to the antigen for a longer period of time [34]. Depot formation is one mechanism by which aluminum salts are thought to work [183]. However, the aluminum depot effect has been challenged, since it has been shown that the antigen in the injection site, absorbed onto aluminum phosphate, was eliminated rapidly within a few hours after injection [184,185]. An adjuvant based on water-in-oil emulsion formulation, called Complete Freund’s Adjuvant (CFA), was also shown to have a depot function, that ensured a prolonged antigen availability [186]. However, due to toxicity, this adjuvant is not allowed for human use. MF59, another water-in-oil based emulsion, is also thought to act by forming a depot, combined with additional mechanisms [187]. Liposomes are also known to act by the depot effect [161].

##### Recruitment of Immune Cells

Adjuvants are known to create a local pro-inflammatory response at the injection site, which leads to the recruitment and activation of immune cells. 

After the idea that aluminum functions by forming a local depot was brought down, different kinds of mechanisms of action came to light. One of them is the recruitment of immune cells. Aluminum salts are known to cause the infiltration of immune cells at the injection site. Most commonly, these are polymorphonuclear cells, like eosinophils, monocytes, neutrophils, dendritic cells, natural killer (NK) cells and NKT cells [188,189]. MF59 is also known to mediate its effect by recruiting immune cells at the injection site. Neutrophils are the first cells to be recruited and are the ones highest in number. Monocytes, eosinophils, macrophages and dendritic cells are also recruited [190,191]. AS03 is another oil-in-water emulsion, authorized for use in 2009 [192]. It has been shown to enhance the recruitment of neutrophils, eosinophils and monocytes at the injection site. These cells then take up the antigens and are responsible for their trafficking to the draining lymph nodes [193,194]. AS04, an adjuvant composed of a TLR4 agonist, MPL and an aluminum salt, is also shown to increase the number of dendritic cells and monocytes in draining lymph nodes [195]. Cationic liposomes (DDA/MPL), when injected intraperitoneally, showed an increased influx of neutrophils, monocytes, macrophages and NK cells [196]. CAF01, a different cationic liposome, has increased the recruitment of monocytes to the site of injection as well as the trafficking to draining lymph nodes [197]. 

##### Enhanced Antigen Uptake and Antigen Presentation

A very important aspect of the activation of adaptive immune response is the efficient uptake of antigens by APCs, and the following presentation by MHCs receptors [34]. Aluminum hydroxide was shown to increase the antigen uptake by dendritic cells and enhance the level and duration of antigen presentation [198,199]. This is possibly due to the decreased degradation rate of the internalized antigen [200]. MF59 is also believed to enhance the antigen uptake, after recruiting immune cells to the injection site [201]. The recruitment of a variety of APCs, together with the increased antigen uptake, leads to a more competent immune response [202]. CpG oligodeoxynucleotides (CpG ODNs), are known to be potent TLR9 agonists, and by this they enhance the humoral and cellular immune responses. They can promote the activation of APCs and facilitate the expression of MHC receptors, which further improves antigen presentation [192].

##### Cytokine and Chemokine Induction

The induction and upregulation of cytokines and chemokines is also known as immunomodulation. Immunomodulation refers to the ability of adjuvants to modify the cytokine network [180]. Cytokines are small, secreted proteins that have an impact on the interactions between cells. Chemokines are cytokines with chemoattractant properties. Both of them can have a proinflammatory or an anti-inflammatory effect [203]. Immunomodulation done by adjuvants can have a stimulatory effect in the upregulation of the entire immune system, however, it usually results in the upregulation of some cytokines and downregulation of others [180].

Mosca et al. demonstrated that alum, MF59 and CpG-ODN can modulate a cluster of genes encoding cytokines, chemokines, innate immune receptors, adhesion molecules and interferon-induced-genes [204]. MF59 seems to be a powerful adjuvant due to its ability to stimulate different chemokine secretion, like CCL2, CCL3, CCL4, CCL5 and CXCL8, from different immune cells. This in turn induces leucocyte recruitment, antigen uptake and activation of the adaptive immune system [201,205,206]. AS03 is also known to stimulate the immune system by the activation of proinflammatory cytokines and chemokines. Upregulation of CCL2, CCL3, and CCL5 seems to be correlated with ASO3 activity [193,194]. CpG-ODNs, which represent strong TLR9 agonists, are recognized by endosomal TLR9. This results in the activation of a signaling cascade, which ultimately ends in the upregulation of proinflammatory cytokines (IL-6, IL-12, IL-18, and TNFα) [207,208,209]. Aluminum-containing adjuvants induce the secretion of cytokines and chemokines by activating NOD-like receptors (NLRs) through direct stimulation of the NLRP3/NALP3 inflammasome complex [210,211,212]. 

### 5.4. Nanoparticles as Vaccine Delivery Vehicles

Nowadays, remarkable efforts have been made in the development of new vaccines as well as in the improvement of already existing ones. Next to the traditional inactivated, live attenuated, virus-vectored and subunit vaccines, stand the newly emerging technologies, such as nanoparticle vaccines [153]. In order for humoral and cell-mediated immunity against infectious diseases to be obtained, the development of effective vaccines together with a suitable delivery system is of paramount importance. In this regard, nanocarriers are of particular interest in the field of vaccines as well as immunotherapy, since they can improve the vaccine efficacy and delivery, and they can help in achieving the desired immune response. Nanocarriers improve the efficacy, they are protecting the antigens from proteolytic degradation, they control the release profile and facilitate the presentation of antigens to APC, their uptake and processing [35,36]. 

The interaction of nanoparticles with the immune system is usually dependent on their physicochemical properties (size, size distribution, shape, surface charge etc.), and they are usually perceived as a stranger or danger signal by the immune system. This occurs even when the nanoparticles are not used as carriers for antigens, i.e., as vaccines [36]. They usually come in contact with the innate immune system first, since these defense mechanisms are enriched at the interface with the external environment. At this point, the nanoparticles are no longer pristine, because they undergo chemical and physical changes once they are “released” in the body. These changes usually refer to the surface changes, due the adsorption of proteins on the nanoparticles, and the formation of a so-called biocorona. The biocorona significantly influences the further interactions of the nanoparticles and the immune system [213]. 

**Figure 6:**
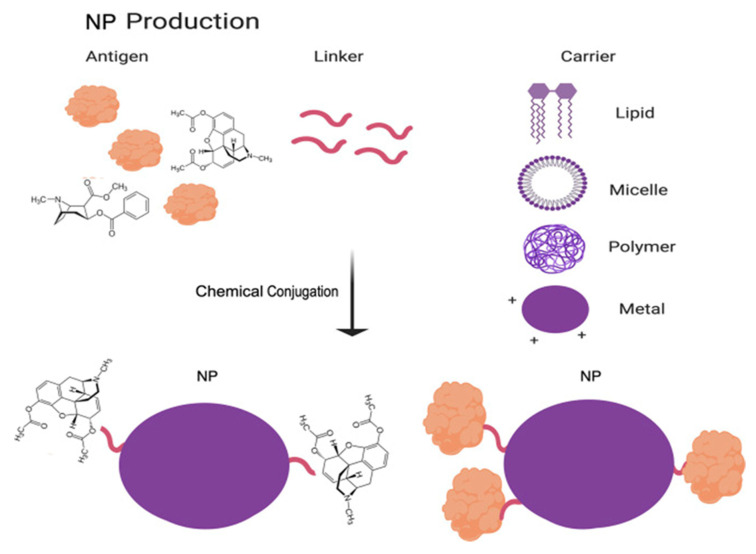
Schematic representation of nanoparticle vaccine production. Reprinted from [153]. CC-BY 4.0 (https://creativecommons.org/licenses/by/4.0/), accessed on 8 February 2021.

When used as delivery systems for vaccines, nanoparticles can be coupled with the antigen of interest in several ways. The antigen can be encapsulated within the nanoparticle, which would offer stability and controlled release. The antigen can also be adsorbed on the surface of the nanoparticles, and in this way, the recognition with surface receptors such as TLRs on APCs can be facilitated [35]. The possible ways of nanoparticle vaccine production are described in Figure 6. 

Nanocarriers composed of metals, lipids, polymers or proteins are gaining more and more attention as potential delivery systems for antigens, which would also offer an adjuvant effect [214].

#### 5.4.1. Inorganic Nanoparticles

Up to now, inorganic nanoparticles have been used mostly as imaging agents, or as photothermal therapy in cancer. However, their characteristics make them attractive candidates in the development of vaccines. Inorganic nanoparticles offer a small particle size, high stability, high drug-loading capacity and a triggered and controlled release profile, which would be ideal for antigen delivery. What is more, inorganic materials can act both as an antigen delivery system, as well as adjuvants [215]. 

Gold nanoparticles (AuNPs) have been applied in many fields so far (such as catalysis, sensing probes and drug delivery). Due to the low toxicity and chemical diversity in terms of shape, size and possibilities for surface engineering, AuNPs can be used in vaccine development [216]. Tao et al. immobilized the extracellular domain of the M2 membrane protein AuNPs and formulated it together with CpG as a TLR-9 agonist, in order to evaluate the immune response against different influenza A subtypes [217]. After receiving the vaccine, mice challenged with H1N1 influenza virus showed high levels of M2-specific antibodies, which resulted in complete protection. Another example where an antigen was conjugated to AuNPs is the work of Safari et al. They have managed to functionalize AuNPs with synthetic tetrasaccharide epitopes from the Streptococcus pneumoniae type 14 capsular polysaccharide [218]. The conjugated polysaccharide induced specific IgG antibodies and activation of memory T-cells in mice. 

Another very attractive delivery platform are mesoporous silica nanoparticles (MSNPs). They are chemically stable, biocompatible and biodegradable. MSNPs can easily incorporate antigens since they have a high surface area due to their porosity [219]. The Soluble Worm Antigenic Preparation (SWAP) antigen was loaded onto MSNPs, which were then used as a vaccine adjuvant against Schistosoma mansoni parasites [220]. Oliveira et al. have showed that the SWAP-loaded MSNPs improved the immunogenicity of the antigen, compared to the use of the antigen with aluminum salts in mice. Furthermore, the antigen specific IgG antibodies were present in mice 112 days after immunization. Guo et al. have developed MSNPs loaded with porcine circovirus type-2 open reading frame (PCV-ORF2) protein, that should act against post-weaning multi-systemic wasting syndrome (PMWS). The nanoparticles demonstrated high antigen loading capacity and slow release, which promoted long-lasting humoral and cellular immune responses [221].

Iron nanoparticles can also be used as vaccine adjuvants, since iron has a role in the initiation of the inflammation process [215]. Neto et al. have synthesized manganese ferrite nanoparticles coated with citrate and modified with Mycobacterium tuberculosis fusion protein. The nanoparticles were applied subcutaneously and intranasally in murine models of tuberculosis, in order to investigate the type of stimulated immune response. Both modes of administration induced a strong immune response. Subcutaneous vaccination induced specific Th1 and CD8+ responses, while intranasal vaccination induced Th1, Th17 and Tc1 responses [222]. Iron nanoparticles have also been coated with silica and loaded with mannose and HBsAg. HBsAg is a surface antigen coming from HBV. Rezaei et al. used these nanoparticles to selectively target dendritic cells (DCs), since they have a high expression of mannose receptors. The nanoparticles showed to be successful in DC-targeting, leading to an increase in the IL-6, TNF-α and IFN-γ gene expression in DCs. In mice immunized with these nanoparticles and increase of cytokine levels was observed [223]. 

It is also worth mentioning that inorganic nanoparticles can also be used in cancer immunotherapy [224]. AuNPs have been coated with ovalbumin antigen (OVA) and CpG as a TLR-9 agonist. The platform has been tested in 4T1 breast cancer model. The nanoparticles are thought to be able to promote a T-cell response directed at the tumor-associated antigen, by displaying the antigen together with MHC class I receptors to CD8+ T-cells. In mice treated with these nanoparticles, there was a noticeable reduction in tumor growth [225]. Silica nanoparticles have also been used in the delivery of OVA and CpG, which also results in reduction of tumor growth [226]. In this case, the studies done in vitro showed enhanced activation and antigen presentation of DCs and increased secretion of proinflammatory cytokines. In vivo, antigen-specific cytotoxic T-cells (CTLs) were activated and suppressed the tumor growth in mice. Iron nanoparticles can also be used in cancer immunotherapy. An interesting example is the design of iron oxide-zinc oxide core-shell NPs that are able to target DCs. In this case, about 95% of the dendritic cells took up the particles, which were then localized in endosomes and lysosomes. In vivo studies showed that the nanoparticles induced an antigen-specific CD8+ T-cell response [224].

#### 5.4.2. Liposomes

Particulate systems, such as liposomes, offer the potential to function as a delivery system for an antigen, but they can also act as adjuvants. This means that liposomes can offer protection for the antigen, enhance its delivery and promote antigen presentation [37]. Liposomes are self-assembling particles, composed of a phospholipid bilayer shell and an aqueous core. Due to their structure, they can be designed to incorporate either hydrophobic antigens (in the lipid bilayer) or hydrophilic antigens (within the aqueous core) [39]. Their potential as antigen delivery systems and adjuvants is influenced by their physicochemical properties (size, charge) as well as antigen location. For example, studies have shown that the administration of smaller particles (100–200 nm) induces enhanced Th2 response, while larger particles (around 600 nm) induce a Th1 response [227]. Furthermore, the liposomal charge influences their adjuvant activity. Cationic liposomes have been proven to promote antigen-binding to their surface, stimulate the interaction with the anionic surface of APCs and promote a strong immune response, compared to neutral or anionic formulations [37]. 

The influenza virus is one of the life-threatening pathogens that need an urgent development of an effective vaccine. As it was mentioned before, liposomes can help in activating the immune system against influenza by enhancing the deposition in draining lymph nodes, increasing the interaction with APCs and by improving the activation of B-cells. Vu et al. have shown that the influenza hemagglutinin (HA) immunogens can be attached to the surface of cobalt-bearing liposomes using microfluidics. The HA-liposomes were successful in eliciting a much higher serum antibody titer in mice and non-human primates compared to the soluble HA used alone [228]. Another example where liposomes have been used to aid antigen delivery and efficacy is the development of a malaria vaccine. In this case, recombinant Pfs25 (a malaria transmission-blocking vaccine antigen candidate) was mixed with liposomes, which resulted in the formation of a particulate antigen. The vaccine seemed to induce long-lived, antigen-specific plasma cells [229]. Tuberculosis is another disease that has been a major problem worldwide. Mansury et al. evaluated the immunogenicity of *Mycobacterium tuberculosis* fusion protein encapsulated in liposomes composed of a cationic lipid and trehalose-6,6′-dibehenate (TBD). TBD is known to stimulate APCs and induce strong Th1 and Th17, which is desirable in tuberculosis immunity, since the activation of Th2 is known to suppress the immune response towards *M. tuberculosis*. The liposomes combined with the fusion protein managed to successfully stimulate Th1 responses in mice [230]. Liposomes can also be combined with other adjuvant molecules to increase the immune response. A TLR9 agonist, known as CpG-ODN, can be linked to liposomes in order to potentiate the antigen stimulus. In one case, CpG-ODN was covalently bound to the *Streptococcus* GBS67 antigen and then electrostatically bound to a cationic liposome. Due to a depot formation, the vaccine managed to induce an increase of functional immune responses against GBS compared to the co-administration of the three single components [231]. Another example where CpG-ODN was linked to liposomes is a vaccine against leishmaniasis, formulated into dissolvable microneedle patches. However, in this case, the inclusion of liposomes weakened the immune response [232]. Besides infectious diseases, cancer is one other disease that can greatly benefit from immunotherapy and vaccination. Cancer vaccines can be used in order to provoke immunity against tumors which are poorly immunogenic. Cationic liposomes have been used for the delivery of mRNA molecules that can encode the desired tumor epitopes and stimulate a T-cell response [233]. Liposomes have also shown to be successful in encapsulating different synthetic long peptides containing a cytotoxic (CD8+) as well as helper T-cell (CD4+) epitope and in inducing tumor specific T-cell responses [234]. 

Nanocarriers such as liposomes have also been used in the treatment of SARS-CoV-2, the virus that caused a pandemic in the beginning of 2020. The recently approved vaccines, coming from BioNTech and Moderna, both contain a mRNA molecule encoding the S-protein of SARS-CoV-2. The mRNA molecule is encapsulated in lipid carriers [30]. Another example is the coupling of synthetic peptides mimicking the N-protein of SARS-CoV-2 onto the surface of liposomes. This vaccine managed to induce a CoV-specific T-cell response [235]. Last but not least, liposomes can be used in the production of a synthetic cell-surface-like competitor to the virus. In this case, liposomes are fused with ACE-2-like membrane proteins. The interaction between ACE-2 receptors on pulmonary cells and the viral spike (S) protein is the one that triggers the infection. In ideal case, the so-called pulmonary-proteoliposomes should be able to competitively bind the viral S protein instead of pulmonary cells [236].

#### 5.4.3. Virus-Like Particles (VLPs)

Virus-like particles are nanosized structures that bare great similarities to viruses that can be helpful in vaccine development. They are made out of viral structural proteins that have the intrinsic ability to self-assemble in particles. Despite being able to “pack” like viruses, VLPs lack a genome and therefore, lack the viral pathogenicity. They are composed of identical protein copies that form capsomeres and can further form icosahedral or helical structures. VLPs vary in size from 20 to 100 nm and offer a repetitive surface structure that renders them highly immunogenic, and therefore, they can be helpful as adjuvants. Due to their size and geometry, they can easily present antigens to MHC I and MHC II surface receptors and activate a strong and lasting B-cell response [38,237].

Recombinant influenza VLPs have been developed as vaccines against the H7N9 virus. The recombinant VLPs morphologically and biochemically resemble the wild-type influenza virus but lack the genetic material. As antigens, they most commonly carry the hemagglutinin antigen (HA) or the viral neuraminidase. After intramuscular or subcutaneous application in mice, the vaccines have shown to induce immunity against the aforementioned antigens [238]. In the approach to develop a more universal influenza vaccine and eliminate the need of an updated vaccine every year, there is a potential to use a more conserved epitope, such as the stem region of HA with VLPs. VLPs produced out of the hepatitis B virus core protein have been used as carriers for the HA stem region and were able to elicit protective immunity [239]. Quan et al. have discussed the development of VLP vaccines against respiratory viruses in a greater detail [240]. The most recent HPV vaccine is also composed of VLPs. In this case, the particles are derived from the major capsid protein, L1, which is not conserved among many HPV types. These vaccines, however, are prophylactic and would not treat existing infections [241]. The highly conserved capsid protein, L2, on the other hand, is more immunogenic, however, it is not capable of self-assembling in VLPs. Nevertheless, it can be displayed on VLPs by chemical conjugation or genetic insertion. It has been shown that VLP-L2 vaccines elicit antibodies with a broad and efficient level of protection against diverse HPV types [88]. VLPs have also been shown to induce immunity as vaccine delivery systems against malaria and arthropod-borne viruses [242,243], and Caldeira et al. have discussed their use as cancer vaccines [244].

In light of the COVID-19 pandemic, caused by SARS-CoV-2, VLPs have been used as tools to study its structural properties as well as potential vaccines. Swann et al. have assembled SARS-CoV VLPs by co-expressing the viral proteins S, M and E in mammalian cells [245]. The M (membrane) and E (small envelope) proteins seem to be essential as structural proteins for the formation and release of SARS-CoV VLPs, and the S (spike) protein forms the spike trimers, which are responsible for receptor binding [246]. Fougeroux et al. have developed so-called capsid-like particles (CLPs) that display the receptor-binding domain (RBD) of the SARS-CoV-2 spike protein. Tested in mice, these particles seem to induce levels of neutralizing antibodies comparable to those found in patients that had recovered from COVID-19 [247]. Furthermore, when encapsulating viral mRNA, VLPs can also be used as a positive control for RT-qPCR detection of SARS-CoV-2 [248].

#### 5.4.4. Biodegradable Polymeric Nanoparticles (NPs)

Due to being capable of drug/antigen delivery and being biodegradable, polymeric NPs have gained much attention. These polymers usually include poly(α-hydroxy acids), poly(amino acids) or polysaccharides, that are able to encapsulate or display antigens on their surface. Polymeric nanoparticles offer a great control over antigen release, and this can be managed through compositional changes in the polymer structure or the use of copolymers. The most commonly used polymers for nanoparticle preparation are poly(lactic-*co*-glycolic acid) (PLGA), poly(lactic acid) (PLA), polyethyleneimine (PEI) etc. [39]. Polymeric NPs are capable of targeting both the innate and the adaptive immune system [249].

PLGA NPs are known to possess intrinsic adjuvant activity. This is most probably due to sustained antigen release and enhanced uptake by DCs. They are also able to increase the expression of MHC class II and activate T-cells. They have been shown to produce higher serum antibodies against ovalbumin or bovine serum albumin, compared to the application of these substances alone [250]. Since PLGA is negatively charged, this could potentially interfere with the adsorption or encapsulation of negatively charged antigens as well as with the interaction with the surface of APC. In this regard, combining PLGA with PEI, which is positively charged, leads to a potent and long-term antigen-specific response [251]. This could be due the capability of PEI to disrupt endosomal membranes in APS by the “proton sponge effect” and release the antigen. It could also potentiate the immune response by activating TLRs and cytokine secretion as well as inflammasome activation [252]. PLGA NPs have also been used as delivery platforms for TLR7/8 agonists in a cancer vaccine. In order for a tumor-specific T-cell response to be elicited, T-cells need to be stimulated by an antigen and a costimulatory molecule by DCs. PLGA NPs were successful to co-deliver tumor-associated antigens (TAAs) and TLR7/8 agonists, such as CpG-ODN, since it can encapsulate both hydrophobic and hydrophilic compounds [253]. When it comes to SARS-CoV-2 therapy, computational simulation design has been used to predict the possibility of incorporating two drugs with different solubility in PLGA NPs. Remdesivir (an antiviral prodrug blocking the activity of SARS-CoV-2-RdRp complex) and lisinopril (an ACE inhibitor) have shown synergism in their anti-SARS-CoV-2 action, and they can be assembled in a remdesivir-PLGA core/lisinopril shell NPs [254]. Chitosan is another commonly used, biodegradable, polysaccharide-based natural polymer that shows immunomodulatory effects and is suitable for mucosal vaccination [255].

#### 5.4.5. Cell-Penetrating Peptides

Cell-penetrating peptides (CPPs) represent a family of cationic and amphipathic peptides, usually not exceeding the length of 30 amino acids. They are famous because of their ease in membrane crossing without causing any harm to the cellular integrity. Besides having a plethora of evidence regarding their success in cargo delivery inside cells, there is still some fog covering their exact internalization mechanisms. Two possible ways have been reported in literature so far, and these include direct translocation through the cellular membrane (passive uptake) and endocytosis (active uptake). The complexity of these mechanisms is too big for the scope of this review however, it is known that they can be divided in sub-classes, and all of these have been involved in the uptake of known CPPs. They have been reported as successful in the delivery of proteins, peptides and nucleic acids. CPPs are discussed in a more detailed manner in the previous chapters.

**Figure 7:**
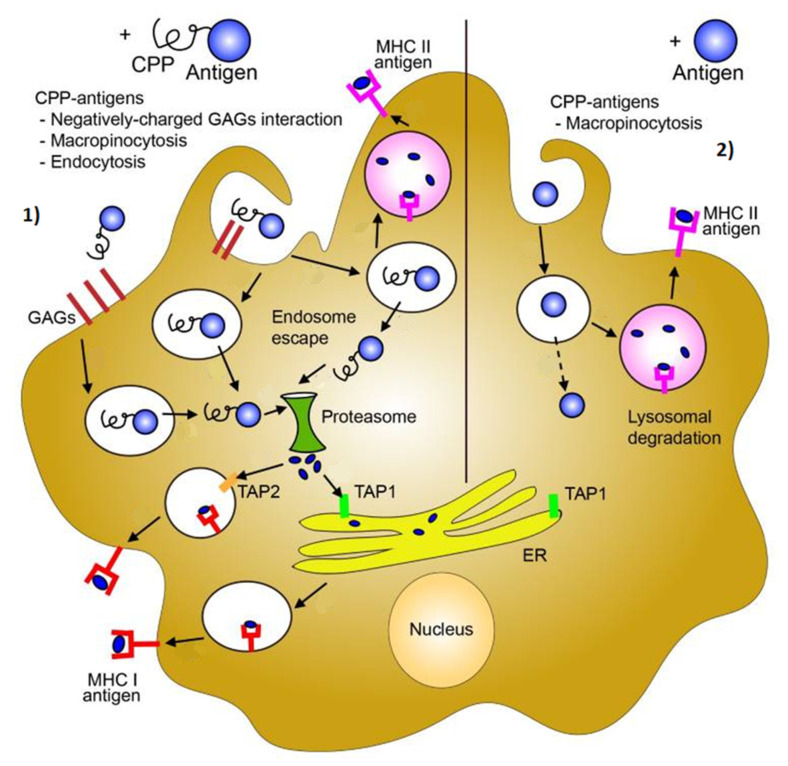
Interaction of cell-penetrating peptides (CPPs) with dendritic cells (DCs), when used as delivery systems for antigens. (1) The CPP–antigen complex interacts with the negatively charged glycosaminoglycans (GAGs) on the surface of dendritic cells. This can trigger the uptake of the complex, usually by endocytosis. When taken up by the cells, the complex is most likely to end up in endosomes. Thanks to the structure of CPPs, the complex can escape the endosomal pathway. After endosomal escape, the complex is degraded in the proteasome, and the antigen is transported to the cell surface by vesicles containing MHC class II receptors. Another possibility is that the antigen is transported though the endoplasmic reticulum-Golgi pathway, and afterwards presented on the cell surface by an MHC class I receptor. If the complex remains in the endosomes, however, it is a subject of lysosomal degradation, after which the antigen is presented on the cell surface by an MHC class II receptor. (2) The free antigen can also undergo endocytosis and enter the cytoplasm of DCs, thus enter the endosomal pathway. Since free antigens are less likely to escape the endosomes, they are subjected to lysosomal degradation, and then presented on the cell surface by MHC class II receptors. Reprinted and edited from [257]. CC BY-NC 4.0 (https://creativecommons.org/licenses/by-nc/4.0/), accessed on 14 March 2021.

With regard to vaccine development, this question is important, because the mechanism of uptake oftentimes has the pivotal role in deciding what type of immune response will be elicited. Besides this fact, the charge, conformation, cargo and concentration play a role in the immunogenicity of CPPs [40]. When it comes to APCs and antigen delivery, CPPs can deliver antigens via both pathways mentioned. Nonendocytic delivery of the antigen will result in antigen processing into short peptide fragments by the proteasome, and these will then be recognized and presented to MHC I molecules, activating cytotoxic CD8+ T-cells. On the other hand, if the CPP–antigen complex is taken up by cells in an endocytic manner (in this case by phagocytosis), it will probably end up in endosomes. Here, it is very likely that, through activating TLRs, MHC II molecules will be induced. This is followed by the activation of helper CD4+ T-cells and the induction of humoral immunity [256]. Tat, MPG, polyarginines and penetratin are just some of the well-known CPPs able to function as antigen carriers. Figure 7 gives a description of the mechanisms of actions of CPPs as delivery systems for vaccines.

Tat-based constructs are very popular for gene delivery, especially for the delivery of DNA. However, besides being a carrier for DNA molecules that code for antigens, Tat can also carry DNA molecules used as adjuvants. Tang et al. have developed a fused HPV E7 oncoprotein (acting as an antigen) and Tat conjugate, where GM-CSF DNA was used as an adjuvant. The nanoparticles were able to eradicate tumors in mice [258]. Tat has also successfully improved the mucosal vaginal delivery of a HIVgag p24 gene. Here, Tat was complexed with a recombinant adenovirus to serve as a carrier for an HIV vaccine [259]. A vaccine candidate against hepatitis B virus containing Tat has also been designed. The fusion of Tat, hepatitis B core antigen (HBcAG) and maltose binding protein (MBP) resulted in a MBP-HBcAG-Tat fusion protein, that strongly enhanced IgM antibody production in mice [260]. Furthermore, in an attempt for developing an anti-tuberculosis vaccine, the recombinant fusion protein of the antigen Ag85B gene and Tat was expressed in *E. coli*. Ag85B is known to induce strong protective response against *M. tuberculosis*. Mice immunized with this fusion protein produced high Ag85B specific IgG antibodies and cytokines [261].

MPG, an amphipathic CPP designed based on SV40 nuclear localization sequence and the fusion sequence of HIV glycoprotein 41, has also been used as an antigen delivery system. Saleh et al. have designed an MPG-based anti-HPV system. This is composed of the MPG peptide and a plasmid encoding the gene for antigen E7. The complex managed to regress the growth of a tumor caused by the virus in mice [262]. In the effort to develop a carrier system for an HIV-1 vaccine, MPG was compared to histidine-rich nona-arginine (HR9) regarding the efficacy of noncovalent delivery of the Nef antigen into cells. MPG showed much higher efficiency in delivery than HR9 and induced a stronger Th1 cellular immune response in a murine model [263]. Similar results were obtained in a study where a DNA construct encoding multiple HIV epitopes was designed. The designed DNA included genes for nef-vpr-gp160-p24 epitopes and was complexed with MPG through non-covalent interactions. The complexes were able to interact with cells and induce humoral and cellular immune responses in vivo [264]. MPG was also used as a delivery system for the hepatitis C virus (HCV) antigens. In this case, two DNA constructs encoding HCV core and coreE1E2 genes were complexed with MPG, and then the efficacy of the complexes was compared to that of the antigens used alone in Balb/c mice. Mice immunized with the complexes generated a mixture of IgG1 and IgG2 antibodies as well as increased IFN-γ production [265]. Furthermore, MPG was used to assist antigen cross-presentation and increase the tumor immune response to a tumor vaccine. Liu et al. reported the production of a nanovaccine, produced by encapsulating ovalbumin as a model antigen (OVA) chemically modified with MPG, into PLGA nanoparticles. The complex eased the release of the antigen in the cytosol of dendritic cells, and it promoted their maturation. Furthermore, is was able to activate tumor specific T-cells and suppress the tumor growth compared to free or unmodified OVA [266]. 

Polyarginine is a CPP designed based on the Tat sequence and exhibits similar translocation properties. Besides being used as a drug carrier, it can also be used as a vaccine delivery system. Wang et al. have developed a vaccine carrier peptide Cys-Trp-Trp-(Arg)_8_-Cys-(Arg)_8_-Cys-(Arg)_8_-Cys, which was used to form nanocomposites with OVA by electrostatic interactions. The complexes were stabilized by redox-responsive disulfide bonds, which are supposed to be reduced by intracellular glutathione. The arginine residues improved the uptake of the complex in APCs, where the antigen was later rapidly released and was able to induce potent CD8+ T-cell immunity [267].

Penetratin, also known as the antennapedia transduction sequence, is a natural CPP derived from the homeodomain protein of *Antennapedia*. It has been used for enhancing tumor antigen percutaneous delivery. Penetratin was linked to OVA and was used for epicutaneous immunization in mice. This resulted in the production of a high level of OVA-specific CD8+ T-cells compared to the mice treated with OVA alone [268].

### 5.5. Protamine in Vaccine Development

Protamine is a highly basic peptide that belongs to the family of cell-penetrating peptides. It is highly specialized in replacing histones during the final condensation of DNA in sperm. Its structure is rich in arginine residues, which are responsible for the cationic charge [41]. Furthermore, the arginine sequence allows protamine to spontaneously associate with negatively charged molecules, such as nucleic acids, in vitro. It is most commonly used as a transfection agent for nucleic acids (DNA, mRNA, miRNA, siRNA) and oligonucleotides (antisense-ODNs, CpG-ODNs) [26]. Due to the guanidinium group found on the arginine residues, protamine can easily interact with cellular membranes by forming bidentate bonds and drive the uptake of the cargo inside the cell. Thanks to the nuclear localization signals in its sequence, protamine is effectively taken to the cell nucleus, which is why it represents a great carrier for DNA molecules. However, the cargo can be released in the cytoplasm as well, which facilitates the use of protamine as a carrier for RNA molecules, that need to be released in the cytoplasm in order to be effective [269]. 

**Figure 8:**
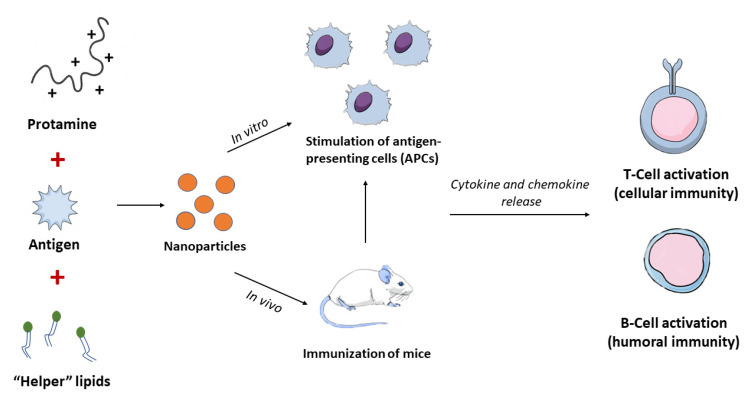
Mechanism of action of protamine vaccines. When protamine is complexed with antigens, it forms NPs that can be used as delivery systems for the antigens. Whether applied in vitro or in vivo, protamine vaccines stimulate the antigen-presenting cells (APCs), such as dendritic cells, which in turn start releasing cytokines and chemokines. These signaling molecules further activate the humoral immunity (B-cells which produce antibodies) and cellular immunity (T-helper cells and cytotoxic T-cells).

As a part of the CPP family, protamine also offers the possibility to be used in the development of vaccines. It can be used as a delivery system for antigens, as a DNA/RNA condensation agent together with different types of nanoparticles such as liposomes, as an adjuvant due to some intrinsic ability to potentiate the immune response and, last but not least, as a gene carrier for ex vivo stimulation of APCs, which are supposed to be used as vaccines themselves. Figure 8 gives a brief description of the mechanisms of action of protamine vaccines, which are discussed in further detail in the following text.

Protamine’s role as an adjuvant and antigen delivery system has been explored in the design of so-called “danger signals”. Basically, “danger signals” are molecules with immunostimulatory properties that are commonly found as patterns on the surface of pathogens or represent nucleic acids, able to stimulate surface, intravesicular and cytosolic proteins. One type of receptors for these “danger signals” are the already mentioned Toll-like receptors (TLRs). A strong ligand for TLRs, especially TLR-7 and -8 is single-stranded RNA (ssRNA). When in touch with TLRs, ssRNA can induce a broad range of immune responses. Protamine is used to stabilize ssRNA thanks to electrostatic interactions and to protect it from nucleases. In this way, particles are formed, which vary in size and show a difference in the stimulation of TLRs. It has been shown that particles smaller than 450 nm trigger plasmacytoid dendritic cells and secretion of interferon α. These are of great interest for anticancer and antiviral therapies. On the other hand, larger particles activate monocytes and production of TNF-α [270]. 

Scheel et al. have also combined mRNA and protamine in order to form stable nanoparticles which would have immunomodulatory properties. The complex was tested in vivo by injection into a mouse ear pinna, and it showed to trigger T and B-cell immune responses directed against the antigen encoded by the mRNA molecule. Here it was demonstrated that TLRs are involved, since protamine-mRNA complexes served as danger signals. TLR-1, -7, and -8 might be involved in the recognition of protamine-mRNA complexes and further activation of DCs, monocytes, NK cells, granulocytes and B-cells [41]. 

Protamine has been sought after in the development of nanocapsules for antigen delivery [43]. Here, the model antigen is H1N1 influenza hemagglutinin (H1). The nanocapsules are composed of an oily core, a protamine shell and pegylated surfactants used to further stabilize the system. The protamine shell is thought to facilitate the interaction and internalization of the nanocapsule within cells and control the release profile of the antigen. In vitro studies showed that the nanocapsules were readily internalized by macrophages, probably due to their positive charge owing to the protamine. To test the in vivo efficacy, BALB/c mice were immunized with two antigen doses of the protamine-nanocapsules, and their effect was compared to one coming from antigen adsorbed on alum. The initial antigen response activated by the nanocapsules was higher compared to the alum one, however, it started to decrease after 7 weeks. However, one interesting finding was that the immune response reached similar levels regardless of the dose of antigen-loaded nanocapsules used. This could offer the possibility of administering lower antigen doses by using protamine-nanocapsules and eliciting an efficient immune response [43].

CpG-ODNs, as mentioned earlier, are potent TLR agonists. They are known to induce a Th1 response, driven by the stimulation of TLR-9. The activity of CpG-ODNs can be enhanced by the use of protamine nanoparticles, used as their carriers [44]. The use of protamine nanoparticles significantly increased the CpG-ODN-mediated production of interferon-α and stimulated B-cells to secrete high amounts of IL-6. The CpG-ODN-protamine combination has been explored in the design of protective allergy vaccines. Allergen-specific immunotherapy requires numerous antigen doses over a long period of time, in order for IgE-mediated hypersensitivity to be controlled. CpG-ODN, used as an immunostimulatory agent combined with PLGA and protamine, has shown to be effective in inducing Th1-associated IgG2a and stimulates antibody titers in mice correlated with a better allergen protection. The addition of protamine seemed to have improved the effect, probably due to the strong adsorption of CpG on protamine and the following sustained antigen release as a consequence of the strong bond. This would allow the CpG antigen to reach APCs for a longer period of time [45]. Similar results were obtained by Pali-Schöll et al., who complexed protamine with Ara h 2 extracted from raw peanuts and used it as a model antigen. The particles were subcutaneously administered in BALB/c mice, and a favorable increase in Ara h 2-specific IgG2a antibodies was found after immunization, and they were also shown to drive the immune response towards a Th1-meidated immunity. The protamine improved the antigen delivery, probably due to slow and sustained release, which would indicate a fewer antigen doses for successful immunotherapy [46].

Treatment of hepatitis B virus (HBV) is another field where the use of protamine as a vaccine has been explored. Nanocapsules made out of protamine were compared to ones produced out of polyarginine, in order to see which one interacts better with the immune system and would act as a better antigen delivery system [54]. The interaction with the immune system was investigated in the means of cellular uptake assessment, ROS production, complement activation and cytokine secretion. The protamine nanocapsule seemed to be superior in eliciting an immune response compared to polyarginine. This could be due to higher complement activation by protamine nanocapsules and the slightly greater tendency to stimulate cytokine production. 

Furthermore, when tested in vivo as carriers for a model antigen, recombinant hepatitis B surface antigen (rHBsAg), protamine nanocapsules elicited higher IgG levels than the polyarginine ones [54]. Another example where HBV antigen was used in combination with protamine is given by Gonzalez-Aramundiz et al. They have designed nanoparticles composed of protamine and a polysaccharide (hyaluronic acid or alginate) as carriers for HBsAg. The in vitro studies showed an increase in cytokine secretion by macrophages, caused by the nanoparticles. In vivo studies carried out in mice showed that the nanoparticles are able to trigger efficient levels of IgG antibodies against the HBsAg after intramuscular application. Furthermore, the particles were also used for nasal vaccination, and even with this approach, they managed to induce a relatively specific IgG response [47]. This is probably due to the positively charged protamine, that helps in the interaction with the negatively charged nasal mucosa. 

The same group also proved that protamine nanocapsules can have improved thermostability and eliminate the limitations associated with the cold chain storage. The nanocapsules are composed of an oily core with immune-stimulating activity, surrounded by a protamine shell. The nanocapsules successfully associated with rHBaAg. Upon freeze drying, the nanocapsules were able to preserve the activity of the antigen even after 12 months of storage at room temperature [50].

Protamine can be used to stabilize RNA molecules for ex vivo stimulation of primary human dendritic cells (DCs). The formed nanocomplexes were able to stimulate DCs, upregulate maturation markers, MHC receptors and stimulate cytokine production. However, there were some noticeable differences in the immune response that was provoked, coming from different sized particles. Namely, smaller complexes were able to associate better with primary DCs, while CD1c+ DCs associated more with larger complexes. The larger complexes also seemed to induce a higher immune response. This is most probably due to the larger protamine-RNA complexes serving as better ligands for TLR-8 stimulation [56].

Mai et al. have explored the use of cationic liposome-protamine-mRNA complex vaccine as an anti-tumor vaccine [49]. In this case, protamine was used to concentrate and condense the mRNA molecule in the cationic liposomes. This complex showed efficacy in cellular uptake in vitro, a strong capacity to stimulate the maturation of dendritic cells and an induction of an anti-tumor response. What is more, this complex offers the possibility of intranasal administration and anti-tumor vaccination through the nasal mucosa. 

Mannosylated protamine sulfate (MPS) has been used as a DNA carrier in order to improve transfection efficacy and induce anti-tumor response [48]. Anti-GRP DNA vaccine was used as a model antigen and was condensed by MPS into nanoparticles. The nanoparticles improved the antigen delivery into macrophages probably due to the abundance of mannose receptors on their surface, which aided in the receptor-mediated endocytosis of the particles. The particles were localized closely to the nucleus, which is in tune with protamine localization due to its nuclear localization sequences. After intranasal administration in mice, a significant response in GRP specific antibodies was observed. 

Fotin-Mleczek et al. used a two-component mRNA-based tumor vaccine as an approach in cancer immunotherapy. The vaccine is supposed to support both antigen expression and immune stimulation mediated by TLR-7. This vaccine is composed of free mRNA and protamine-complexed mRNA. It was shown that the vaccine induces balanced immune responses, including B and T-cell immunity. In vivo studies proved that the two-component mRNA vaccine elicits a strong antitumor response against OVA-expressing tumor cells in a prophylactic and in a therapeutic setting [42].

When it comes to protamine vaccines being investigated in clinical settings, Weide et al. gave an overview of the outcomes of direct injection of protamine-protected mRNA in metastatic melanoma patients [271]. They have proven that the injection of protamine-protected RNA is safe and in the treated patients it had a significant impact on the frequency of immunosuppressive cells. This would mean that there was a noticeable decrease in Treg cells, which are usually correlated with blocked immune responses in cancer patients. An increase in the anti-tumor T-cells was also achieved. Furthermore, a promising clinical outcome was observed in only 1 of 7 patients with measurable disease. These findings should undergo further investigation in order for the impact of the therapeutic concept to be verified [271]. Another study in clinical settings (phase Ib) was done by Papachristofilou et al. [272]. They have investigated the effect of a protamine-formulated mRNA vaccine in cancer immunotherapy, with a mRNA molecule encoding six non-small cell lung cancer-associated genes. Combined with local radiation, the vaccine was evaluated in patients with stage IV non-small cell lung cancer. The obtained results show that the treatment was well tolerated in all of the patients, with most of the adverse effects being injection site reactions and flu-like symptoms. Furthermore, the vaccine induced antigen-specific immune responses in the majority of the patients. The results suggest that this type of mRNA-based immunotherapy can be further investigated for the combined use together with immune checkpoint inhibitors [272].

The ongoing global COVID-19 pandemic has highlighted the need for technologies that allow rapid development of human vaccines. Protamine is a peptide that offers the opportunity for development of a mRNA-based vaccine against SARS-CoV-2. The preclinical data obtained by Petsch et al. and Schnee et al. showed promise in the use of protamine for successful delivery of antigen-coding mRNA [273,274]. The former tested the protective efficacy of protamine-mRNA vaccines against influenza A infection, while the latter used protamine as a carrier for rabies virus glycoprotein (RABV-G) encoding mRNA. In both cases, the vaccines induced long and balanced humoral and cellular immunity. This, together with the results obtained by Alberer et al. [275] regarding the immunogenicity and safety profile of a protamine-mRNA rabies vaccine in a phase 1 clinical trial, is the background behind CureVac’s idea to use protamine as a carrier for mRNA encoding the SARS-CoV-2 spike (S) protein [276]. However, this idea was quickly abandoned, and lipid nanoparticles (LNPs) were used for complexing the mRNA, instead of protamine. This could owe to the fact that during the clinical trial described by Alberer et al., a high enough antibody titer was achieved only when the protamine-mRNA vaccine was administered by needle-free devices. The intradermal or intramuscular application by using a needle-syringe did not produce a satisfactory level of antibodies [275]. The need for rapid vaccination using conventional and well-known methods of vaccine administration could be one reason why CureVac stopped the development of protamine-mRNA vaccines. However, the protamine-mRNA vaccine developed by Alberer et al. showed a good stability profile in different conditions, and with the possibility of needle-free application, it represents a promising candidate for the development of temperature-stable, safe and effective vaccine. 

## 6. Conclusions

Nanotechnology is the up-and-coming trend in medicine. Nanoparticulate systems in particular are of great interest, since they offer the advantage of better drug stability, controlled release profile, and targeted drug delivery. What makes nanotechnology even more sought after is the possibility that it offers for the delivery of novel therapeutic molecules, such as proteins, peptides, and nucleic acids. During the COVID-19 outbreak, we became witnesses of the importance of this filed in today’s medicine, since most of the modern vaccine design is based on nanoparticles as delivery systems for antigens.

Protamine is a highly basic peptide, and it is a part of the cell-penetrating peptide family. It is frequently used in therapy as a heparin antidote. However, protamine has a special use in the nanotechnology field too. Thanks to its arginine sequence, protamine is capable of spontaneously associating with negatively charged molecules, such as nucleic acids (DNA, mRNA, siRNA, miRNA), or oligonucleotides (such as CpG oligonucleotide) forming nanoparticles, so-called proticles. Due to its ease in interaction with the cell membrane, protamine is used as an agent that can deliver its cargo in the cytoplasm, or take it to the nucleus. So far, there are numerous publications regarding the use and efficacy of protamine as a transfection system. Whether used for the delivery of DNA to the nucleus, or mRNA in the cytoplasm, protamine has proven to be effective in protecting the cargo molecule from enzymatic degradation, improving its uptake inside the cells, and therefore, improving the desired therapeutic effect. Furthermore, the efficacy can be improved by functionalizing or derivatization of the protamine-nucleic acid complexes, using different targeting or stabilizing moieties.

The aforementioned advantages that protamine offers as a delivery system make it rather appealing for use in the development of vaccine delivery systems. Protamine can be used for the delivery of antigen molecules, as a DNA/RNA condensation agent together with other types of nanoparticles, as an adjuvant due to some of its intrinsic abilities to stimulate the immune response, or as a gene carrier in the ex vivo stimulation of APCs, when they are supposed to be used as vaccines in cell-based therapies. The successful use of protamine has already been published in several articles covering vaccination against infectious diseases and cancer. It has been proven that protamine, when combined with antigen-encoding nucleic acids, improves and enhances the immunogenic activity of the antigen. This is probably due to the sustained release profile, that ensures a longer exposure time of the immune system to the antigen. Besides, the efficacy against infectious disease and cancer being proven in numerous in vitro studies done on cell models, or in vivo studies in animal models, protamine has also shown to be effective in the treatment of cancer in the clinical settings. What is more, protamine offers the possibility of mucosal vaccination, as well as the development of a vaccine that would have increased thermostability, and thus, reduce the need of the cold chain storage. This is a great advantage, especially in urgent settings, such as the current COVID-19 pandemic. 

Having in mind the advantageous properties of protamine as an excipient in pharmaceutical preparations, one can state that protamine offers a plethora of possibilities for application in different fields. Thus, protamine represents an exceptionally interesting peptide that should be considered in future research work. 

## Figures and Tables

**Table 1:** Overview of the application fields of protamine.

Application Field	Protamine and NPs Applied	References
Diabetes therapy	Protamine is applied in insulin preparations to form protamine-zinc-insulin complexes as well as Protamine Hagedorn insulin (NPH) in order to prolong the insulin effect.	[10,11]
Heparin antagonist	Protamine free base, protamine chloride and Protamine sulfate are applied as antidote against the anticoagulation effect of negatively charged heparin for example in cardiac surgery.	[12,13,14,15]
Nanopharmaceuticals	Protamines are noninvasive cell penetrating peptides, showing the ability to target drugs to specific molecules within the cells and form nanoparticles by self-assembling with negatively charged macromolecules. All kinds of (derivatized) protamines (free base, chloride, sulfate, low molecular weight) are forming nanoparticles. Modifications with human serum albumin, polyethylene glycol, citric acid, secretoneurin or packing oligonucleotides (ODN) in solid lipid nanoparticles or liposomes were performed.	[25,27,29,51,52,53]
Vaccines	Protamine, used as a carrier for antigenic RNA molecules, in the form of nanoparticles or nanocapsules, can be used as a vaccine and adjuvant. The fields of application include infective diseases, as well as cancer.	[41,43,49,54,55,56]

## Data Availability

Not applicable.
